# Elucidating the picocyanobacteria salinity divide through ecogenomics of new freshwater isolates

**DOI:** 10.1186/s12915-022-01379-z

**Published:** 2022-08-08

**Authors:** Pedro J. Cabello-Yeves, Cristiana Callieri, Antonio Picazo, Lena Schallenberg, Paula Huber, Juan J. Roda-Garcia, Maciej Bartosiewicz, Olga I. Belykh, Irina V. Tikhonova, Alberto Torcello-Requena, Paula Martin De Prado, Richard J. Puxty, Andrew D. Millard, Antonio Camacho, Francisco Rodriguez-Valera, David J. Scanlan

**Affiliations:** 1 Evolutionary Genomics Group, Departamento de Producción Vegetal y Microbiología, Universidad Miguel, Hernández, San Juan de Alicante, Alicante, Spain; 2 National Research Council (CNR), Institute of Water Research (IRSA), Verbania, Italy; 3 Cavanilles Institute of Biodiversity and Evolutionary Biology, University of Valencia, E-46980 Paterna, Valencia Spain; 4 Department of Zoology, University of Otago, Dunedin, New Zealand; 5 Instituto Tecnológico de Chascomús (INTECH), UNSAM-CONICET, Av. Intendente Marino Km 8,200, (7130) Chascomús, Buenos Aires, Argentina; 6 Instituto Nacional de Limnología (INALI), CONICET-UNL, Ciudad Universitaria - Paraje el Pozo s/n, (3000), Santa Fé, Argentina; 7 Department of Environmental Sciences, University of Basel, Basel, Switzerland; 8 Limnological Institute, Russian Academy of Sciences, P.O. Box 278, 664033 Irkutsk, Russia; 9 School of Life Sciences, University of Warwick, Coventry, CV4 7AL UK; 10 Department of Genetics and Genome Biology, University of Leicester, Leicester, LE1 7RH UK; 11 Moscow Institute of Physics and Technology, Dolgoprudny, 141701 Russia

**Keywords:** *Synechococcus*, *Cyanobium*, Freshwater, Marine, Brackish, Salinity divide, Genomics

## Abstract

**Background:**

Cyanobacteria are the major prokaryotic primary producers occupying a range of aquatic habitats worldwide that differ in levels of salinity, making them a group of interest to study one of the major unresolved conundrums in aquatic microbiology which is what distinguishes a marine microbe from a freshwater one? We address this question using ecogenomics of a group of picocyanobacteria (cluster 5) that have recently evolved to inhabit geographically disparate salinity niches. Our analysis is made possible by the sequencing of 58 new genomes from freshwater representatives of this group that are presented here, representing a 6-fold increase in the available genomic data.

**Results:**

Overall, freshwater strains had larger genomes (≈2.9 Mb) and %GC content (≈64%) compared to brackish (2.69 Mb and 64%) and marine (2.5 Mb and 58.5%) isolates. Genomic novelties/differences across the salinity divide highlighted acidic proteomes and specific salt adaptation pathways in marine isolates (e.g., osmolytes/compatible solutes - glycine betaine/*ggp/gpg/gmg* clusters and glycerolipids *glpK*/*glpA*), while freshwater strains possessed distinct ion/potassium channels, permeases (aquaporin Z), fatty acid desaturases, and more neutral/basic proteomes. Sulfur, nitrogen, phosphorus, carbon (photosynthesis), or stress tolerance metabolism while showing distinct genomic footprints between habitats, e.g., different types of transporters, did not obviously translate into major functionality differences between environments. Brackish microbes show a mixture of marine (salt adaptation pathways) and freshwater features, highlighting their transitional nature.

**Conclusions:**

The plethora of freshwater isolates provided here, in terms of trophic status preference and genetic diversity, exemplifies their ability to colonize ecologically diverse waters across the globe. Moreover, a trend towards larger and more flexible/adaptive genomes in freshwater picocyanobacteria may hint at a wider number of ecological niches in this environment compared to the relatively homogeneous marine system.

**Supplementary Information:**

The online version contains supplementary material available at 10.1186/s12915-022-01379-z.

## Background

Picocyanobacteria are the smallest photoautotrophs inhabiting aquatic systems and are key contributors to total oxygen production and CO_2_ fixation on our planet [1, 2]. Their global distribution encompasses marine, freshwater, and brackish ecosystems where they are major primary producers [3–7]. Three main genera exist: *Prochlorococcus*, *Synechococcus*, and *Cyanobium*. While *Prochlorococcus* is absent from brackish and freshwater environments, *Synechococcus* and *Cyanobium* have colonized aquatic ecosystems in virtually all regions of the globe [8–10]. *Prochlorococcus* shows the smallest cell sizes (~0.5–0.8 μm length) [4], which, given their large surface area/volume ratios and hence high nutrient acquisition capacity, is consistent with their abundance in the vast oligotrophic ocean gyres [9]. *Synechococcus* and *Cyanobium* cells are typically larger (1–2 μm) and have the potential to form microcolonies, particularly in freshwater and marine coastal areas [10–13].

Over recent decades there has been a substantial effort to study picocyanobacteria using a combination of ecological and genomic approaches. This “ecogenomics” perspective has largely focused on marine representatives with the first *Prochlorococcus* and *Synechococcus* genomes sequenced nearly two decades ago [14–18], extensively characterized further in an ecological context [19], and subsequently reassessed with a new input of closed genomes [20, 21]. Such work has provided a plethora of information including defining the core and flexible genome of these organisms as well as providing detailed insights into the molecular basis of niche adaptation in these genera [16, 21, 22]. Other genomic studies have explored in detail the phycobilisome (PBS) antenna complexes and chromatic adaptation capacity of marine *Synechococcus* [19, 23–25] as well as more general picocyanobacterial physiological attributes like mixotrophy [26] and CO_2_ concentrating systems [27]. Such genomic information is now publically available in specific searchable databases like Cyanorak [28]. In contrast, genomic studies of brackish and freshwater picocyanobacteria have generally lagged behind, with only a few euryhaline representatives from estuarine [29] and brackish [30, 31] habitats. With regard to freshwater strains, some of the most abundant and cosmopolitan representatives were obtained as metagenome assembled genomes (MAGs) [32] or only recently retrieved and sequenced from Spanish reservoirs [33], a volcanic lake in central Italy [34] and South-American, Central European, and Mexican lakes [35].

While the genus *Synechococcus* is known to be polyphyletic [36–39] at the taxonomic level, the majority of aquatic picocyanobacteria (including those newly reported here) fall within a well-defined clade within the cyanobacterial lineage termed cluster 5 [40] in which there are three major sub-clusters (SCs). *Prochlorococcus* forms a separate branch close to most obligately marine *Synechococcus* within SC 5.1, with these latter *Synechococcus* recently proposed as *Ca*. Marinosynechococcus [21]. Various brackish/halotolerant members, initially considered as obligately marine, are present inside SC 5.2 [16, 19–21, 41, 42]. Conversely a few halotolerant strains isolated from the Red Sea [43] and the brackish Black Sea [31] fall within SC 5.1 [21]. Perhaps the most diverse SC, certainly in terms of the geographic origin of isolates and habitat from which they were obtained, comprising halotolerant, euryhaline and many freshwater representatives, is SC 5.2 [30, 32–35]. Members of this SC were recently specifically assigned to the *Cyanobium* genus [21]. SC 5.3 includes both open ocean strains [19, 43] as well as a cosmopolitan group of freshwater strains with *S. lacustris* as the species type [33]. This SC has recently been assigned to the *Juxtasynechococcus* genus [21]. The most recent evidence suggests that there is clear genomic diversification that separates these three SCs [21], with the true origin of planktonic picocyanobacteria suggested to be from benthic marine or freshwater counterparts during the Neoproterozoic (1000–542 Mya) [44–46], although potential transitions between these environments remain to be clarified.

This study was particularly motivated by the paucity of freshwater picocyanobacterial cultures/genomes with the aim to obtain isolates spanning tropical, temperate, and cold natural lakes and reservoirs, ranging from oligotrophic to eutrophic, such that we could specifically disentangle those genomic characteristics spanning the salinity divide when compared to marine counterparts. To enable this, we used all available culture-based marine and euryhaline *Synechococcus* genomes from the Cyanorak database [28] and compared them with a set of previously studied culture-derived freshwater strains within cluster 5, and 58 newly sequenced isolates. We highlight clear differences in genome size, %GC content, shared (core) and flexible genome content, phylogenetic placement, proteomic isoelectric points, gene presence/absence, and metabolic capabilities between marine, brackish, and freshwater strains that span this salinity divide and identify key differences between sub-clusters.

## Results and discussion

### A new picocyanobacterial dataset of freshwater, brackish, and marine strains

**Fig. 1:**
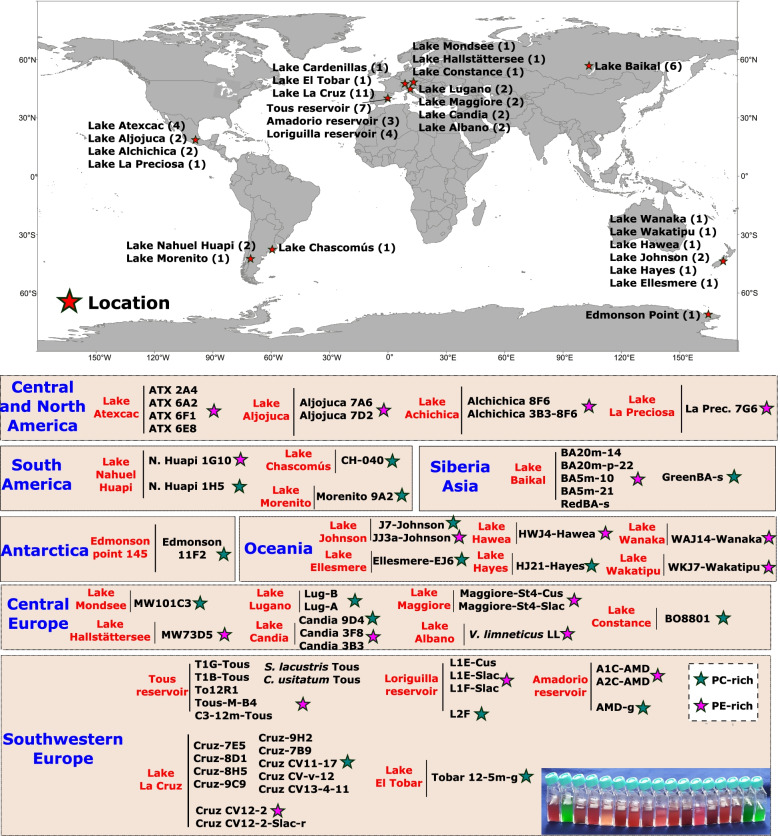
Distribution of the 58 new picocyanobacterial isolates obtained in this study. The number of sequenced genomes is shown in brackets for each location, which is color coded with a red star. Additionally, the names of each isolate, lake, and region of origin are included. Isolates are also coded with green (PC-rich) or pink stars (PE-rich) according to their pigment type composition

To expand the coverage of available freshwater picocyanobacterial genomes, we obtained cultures from various lakes and reservoirs via an isolation campaign spanning more than 5 years, including isolates from several continents (north Asia, west and central Europe, south-east Oceania, Central/North America, South America, and even Antarctica) that reasonably cover the entire globe (Fig. 1). Isolates also covered a range of trophic regimes, from oligotrophic lakes like Lake Baikal (Russia), Lake Nahuel Huapi (North Patagonia, Argentina), Lake Maggiore (Italy), or Tous reservoir (Spain) to mesotrophic habitats such as Amadorio reservoir (Spain), or hypertrophic such as Lake Chascomús (Argentina) and encompassing cold and glacial lakes, temperate reservoirs, and tropical lakes. In total, we sequenced the genomes of 58 new isolates resulting in 32 new species (based on >95% average nucleotide identity, ANI) that greatly expands the current number of complete or near-complete freshwater picocyanobacterial genomes (Additional file 1: Table S1).

Given the phylogeny of the isolates obtained (see “Phylogeny of the new freshwater picocyanobacterial isolates” section below), this work only compares cluster 5 picocyanobacteria, specifically from the *Synechococcus* and *Cyanobium* genera, comprising isolates from different SCs (5.1, 5.2, 5.3) obtained from marine, brackish, and freshwater envionments. A parallel work compares cluster 5 picocyanobacteria (α-cyanobacteria) with other unicellular *Synechococcus*-like strains (β-cyanobacteria) [47]. We used only complete or near-complete genomes (draft) derived from cultures to reduce to a minimum the bias introduced from incomplete genomes derived from metagenomes (MAGs) and single cells (SAGs). A summary of all the main genomic features, origin, and references for the 132 marine, brackish, and freshwater obtained genomes used in this comparison is shown in Additional file 1: Table S1. To clarify the origin of the compared strains, marine, brackish, and freshwater designations were made according to two criteria: (i) their origin and (ii) their tested growth or selection in artificial media. Thus, a marine strain was defined as one isolated from a coastal marine/pelagic open ocean environment that can only grow in marine medium (i.e., >35 g/L NaCl), e.g., *Synechococcus* sp. WH8102 [15] or *Synechococcus* sp. RCC307 [16] from SCs 5.1 and 5.3, respectively. We define as euryhaline/halotolerant those strains that were isolated from brackish/estuarine/coastal systems with intermediate salinities lower than the ocean such as the western Black Sea (18–22 g/L), Pearl River estuary, China (14–35 g/L), or Chesapeake Bay, USA (25–30 g/L). We also define as halotolerant growth in an intermediate salinity medium (5–30 g/L) or across a wide range of salinities, for instance strains BSA11S/BSF8S [30, 31], *Synechococcus* sp. WH5701/RS9917 [16] or LTW-R [29]. Finally, freshwater strains are defined as those that were isolated exclusively using BG-11 medium and have a freshwater pelagic origin, e.g., *S. lacustris* and *C. usitatum* Tous [33], from SCs 5.3 and 5.2, respectively. These latter strains have also been grown at different salinities with optimal growth in BG11 medium and die within days at salt concentrations >3–5 g/L [33]. We acknowledge though that since not all 132 isolates have been analyzed with respect to their growth across a range of salinities, some isolates may be mis-categorized. However, the above definitions provide a modus operandi for moving forward that should be followed until such a time that the precise salinity growth ranges of all picocyanobacteria are known.

### Phylogeny of the new freshwater picocyanobacterial isolates

**Fig. 2:**
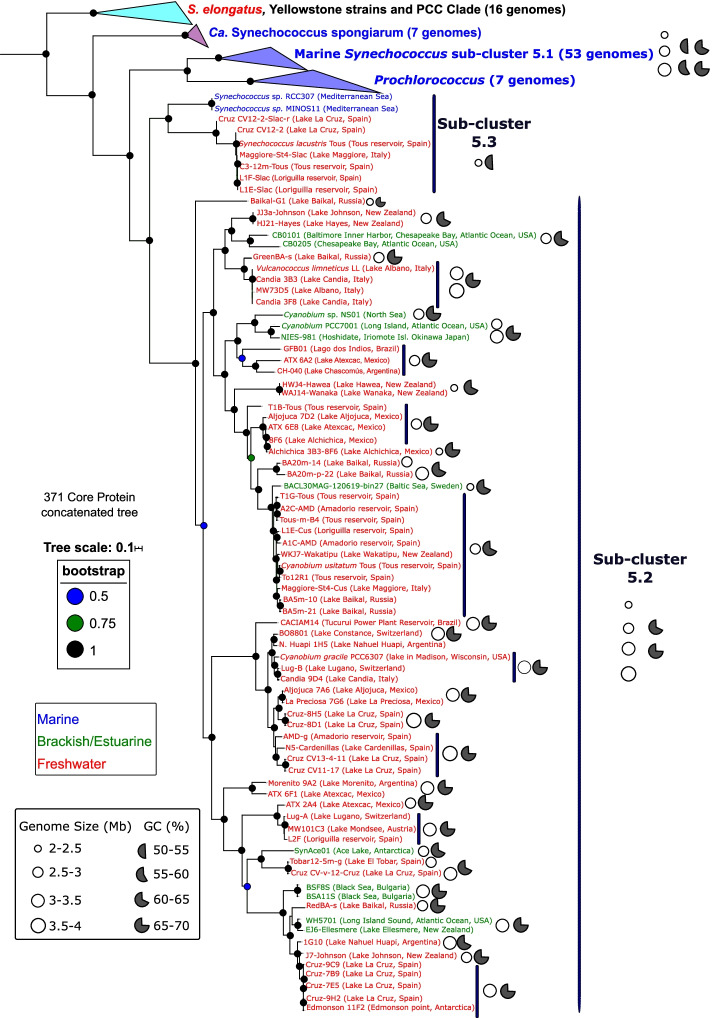
Phylogenetic analysis of all 58 new freshwater picocyanobacterial isolates as well as previously isolated brackish and marine isolates. The phylogeny was rooted at the *S. elongatus* and PCC clade. *Prochlorococcus* and *Ca*. *Synechococcus spongiarum* spp. were added to complete the phylogeny. In total, 365 core universal proteins were used to make the phylogeny (PhyloPhlAn3.0). Bootstrap values >50 are circle color coded. Marine, brackish/estuarine/halotolerant, or freshwater species are indicated in blue, green, and red, respectively. The size of the circle is used as a proxy of the genome size. %GC content is indicated by the filled circle portions

To assess the phylogeny of our newly isolated and sequenced freshwater strains, we constructed a 365-protein concatenated phylogenomic tree including all existing marine, brackish, and freshwater culture-derived cluster 5 picocyanobacterial isolates (Fig. 2). As expected, none of our strains fell inside marine SC 5.1, but rather they all affiliated within either SC 5.2 or 5.3. Our isolates include five new strains within the *S*. *lacustris* clade [33] from various lakes and reservoirs different to the original culture retrieved from Tous reservoir (Spain). Such new isolates came from Lake La Cruz (Spain), Loriguilla reservoir (Spain), and Lake Maggiore (Italy). We also identified a new, closely related species (Cruz CV12-2-Slac-r, albeit only 76-77% ANI to *S. lacustris* genomes), thus delineating two distinct clades with a threshold at 90% ANI (Additional file 2: Fig. S1). All of these strains fall inside SC 5.3 and show very low ANIs compared to all SC 5.1 and 5.2 strains as well as the SC 5.3 Mediterranean Sea isolates MINOS11 and RCC307 (68–72% ANI). Our new SC 5.2 freshwater isolates span a wide diversity (70–95% ANI) and confirm this SC comprises brackish/estuarine isolates as well as freshwater strains. Applying a 90% ANI criteria threshold, we reveal 25 distinct clades inside SC 5.2, forming genetically differentiated species with >90% identity. We also include new strains affiliated to previously studied picocyanobacteria such as *Cyanobium usitatum* [33], *Cyanobium gracile* [39], *Vulcanococcus limneticus* [34], or *Synechococcus* sp. WH5701 [16]. Indeed, isolates closely related to *Cyanobium usitatum* (>90% ANI) were obtained from multiple lakes ranging from cold to temperate as well as from (ultra) oligotrophic to meso-eutrophic conditions. Such new isolates mainly came from cold ultraoligotrophic lakes Baikal (Siberia, Russia), Wakatipu (New Zealand), and oligotrophic Maggiore (Italy) or meso-eutrophic Loriguilla and Amadorio reservoirs (Spain). Hence, the cosmopolitan nature of both *Cyanobium usitatum* and *Synechococcus lacustris* [33] is reflected here having obtained globally distributed new isolates.

### Main genomic features across habitats and sub-clusters

**Fig. 3:**
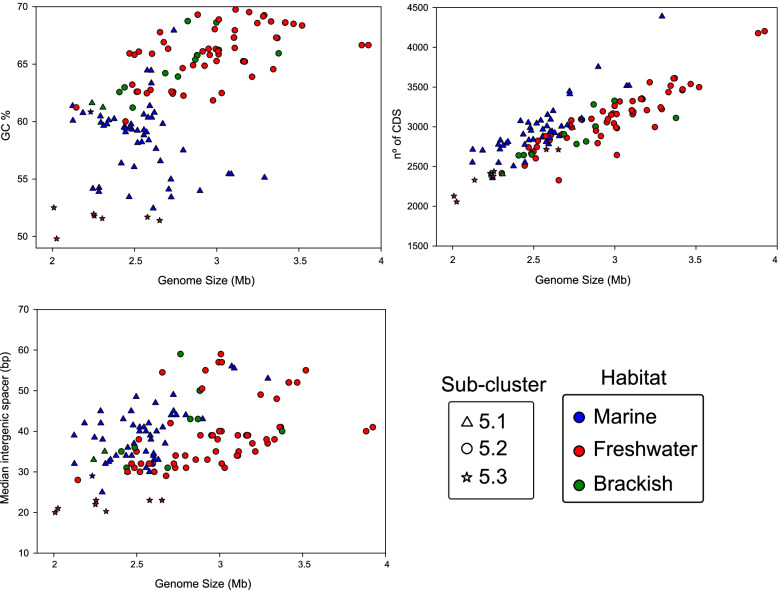
Genome size (Mb) versus %GC content, number of coding sequences (CDS), and median intergenic spacer (bp) plots between picocyanobacterial SCs. Each genome is color coded according to the habitat of origin and shape coded according to the SC to which it belongs

To complement the abovementioned phylogenetics and ANI results (Fig. 2 and Additional file 2: Fig. S1), we plotted genome size, %GC content, and median intergenic spacers from all 132 genomes analyzed (Fig. 3 and Additional file 1: Table S1). We also performed different single pair ANOVA tests between habitats (marine, brackish, freshwater) and SCs (5.1, 5.2, 5.3) to statisticially assess differences in their genome size, %GC content, and median intergenic spacers (Additional file 3: Additional dataset 1). We observed a generally smaller genome size (2.53±0.23 Mb on average) and lower %GC content (58.58±3.19 %) across all isolates from SC 5.1 (*p*-value <0.05) compared to their SC5.2 and SC5.3 counterparts. These mostly open ocean isolates from off-shore oligotrophic waters are the most widespread and possess the smallest *Synechococcus* genomes (<2.5 Mb) encountered so far [16, 19–21]. However, there are a few freshwater strains from SC 5.3 (*S. lacustris* group) that have the lowest %GC content (ca. 52–53%) and smallest median intergenic spacers (20–25 bp) of all the cluster 5 picocycanobacteria (*p*-value <0.05) and are also smaller than 2.6 Mb (Fig. 3, Additional file 1: Table S1), as previously noted [32, 33]. In particular, strains Cruz CV12-2 and Cruz CV12-2-Slac-r have the smallest genome sizes. A handful of freshwater strains from SC 5.2, such as HWJ4-Hawea/ WAJ14-Wanaka (coming from New Zealand lakes) and representatives closely affiliated to the *Cyanobium usitatum* species in SC 5.2 [33], originating mostly from oligotrophic lakes, are also in this size range (<2.6 Mb). All of these SC5.2/5.3 strains are examples of cosmopolitan small genomes that have colonized a wide range of freshwater systems around the globe, from cold ultraoligotrophic to temperate mesotrophic habitats [33] and it is particularly relevant that being small-sized (comparable to marine isolates) they abound in oligotrophic freshwater systems, sharing a similar trophic status to that observed in the ocean.

However, despite the abovementioned exceptions, we generally observed that freshwater and brackish genomes showed a higher %GC content (ca. 64% on average) and larger estimated genome size (average 2.69 Mb for brackish and 2.9 Mb for freshwater strains, with SD of 0.37 and 0.41 Mb, respectively), covering a range between 2 and 4 Mb, a considerably larger range than shown for marine isolates (2.2 to 3.5 Mb) (ANOVA, *p*-value <0.05). Thus, overall genome reduction appears to be much more prevalent in marine representatives.

### The shared and flexible genome of Synechococcus-Cyanobium picocyanobacteria

**Fig. 4:**
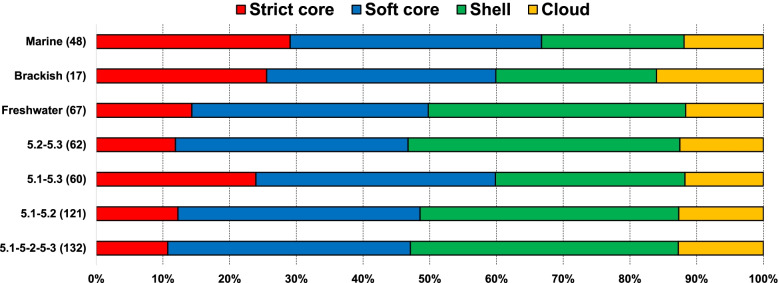
Meta-pangenome analysis of marine, brackish, and freshwater picocyanobacteria from different SCs conducted using the GET_HOMOLOGUES package. The shared genome content is divided into strict core and soft core while the flexible genome is divided into shell and cloud categories. Each category is color coded

To determine how the shared and flexible genome differed between picocyanobacterial SCs and habitats (Fig. 4), we used complete or near-complete (draft) genomes derived from cultures to reduce to a minimum any bias to detect genes belonging to the strict core, soft core, shell, and cloud [48]. Note that this analysis compares a set of microbes belonging to three SCs that span 67–99% ANI, and hence, we are comparing pangenomes of genomically distant populations at the level of genus and family that should not be confounded with strain-level pangenomics. Comparing the meta-pangenome of marine, brackish, and freshwater isolates (Fig. 4) as a whole, we observed a higher percentage of strict core and soft core genes in marine (32% strict core, 41.5% soft core) and brackish (28.95% strict core and 38.8% soft core), compared to freshwater strains (14.3% strict core and 35.4% soft core), consistent with the aforementioned genome reduction in marine picocyanobacteria. Individual comparisons showed that marine strains possessed the highest number of core and soft core genes (1170 strict core genes and 1517 soft core genes) followed closely by brackish representatives (971 and 1303 respectively), but far from freshwater strains (504 and 1240 genes, respectively). These higher values of the persistent/shared genome were also observed when the *Prochlorococcus* and *Synechococcus* pangenome was compared [16]. Overall, our data suggests that freshwater picocyanobacteria have a greater diversity and gene pool compared to their salt-adapted counterparts.

We next repeated these calculations between SCs but this time regardless of salinity origin (Fig. 4). We found relatively high shared gene content (ca. 28 and 37% for strict and soft core, respectively) when comparing the meta-pangenomes of SCs 5.1 (isolates solely of marine/brackish origin) and 5.3 (including both marine and freshwater isolates). This is particularly interesting since SC 5.3, comprising marine strains like RCC307 and freshwater strains like *S. lacustris*, are quite far apart in terms of ANI (67–72%) compared with SC 5.1 and 5.2 isolates. When we included SC 5.2 in the analysis (compared to SCs 5.1 and 5.3), the total number of shared genes was drastically reduced: 10–12% for strict core and 34–36% for soft core, which likely reflects the large genetic diversity present in SC 5.2 encompassing strains spanning the salinity divide and with a wide range of genome sizes (Figs. 2 and 3 and Additional file 1: Table S1).

Analyzing genomes from all three SCs and habitats together (Fig. 4, Additional file 4: Fig. S2A and Additional file 5: Additional dataset 2), we obtained the smallest strict core (351 genes, 10.7% of the total) and soft core (1190 genes, 36.3% of the total) gene set, which represents 47% of the total genomic repertoire. These results led us to determine a strict picocyanobacterial core genome curve, which stabilized at ca. 350 genes, while the meta-pangenome curve comprised >35,000 genes and was far from reaching a plateau (Additional file 4: Fig. S2B). This trend was also observed in a pangenomic study of marine SC 5.1 *Synechococcus* [16]. As expected, >80% of all genes belonging to strict and soft core were all related to amino acid biosynthesis (ca. 7.5 %), protein metabolism (ca. 10–13%), carbohydrates (6.8%), cell division and cell wall biosynthesis (5%), photosynthesis (ca. 2.5%), DNA/RNA metabolism (7%), or fatty acid and lipid metabolism (ca. 3%) (Additional file 4: Fig. S2C). On the other hand, >80% of the genes associated with the shell and cloud categories were labelled as other categories based on SEED, which exemplifies the enormous number of hypothetical and unknown functions in the flexible compartment of these microbes. A list with SEED annotation for all these four pangenome categories is shown in Additional file 6: Additional dataset 3.

### General features of the picocyanobacterial proteome

**Fig. 5:**
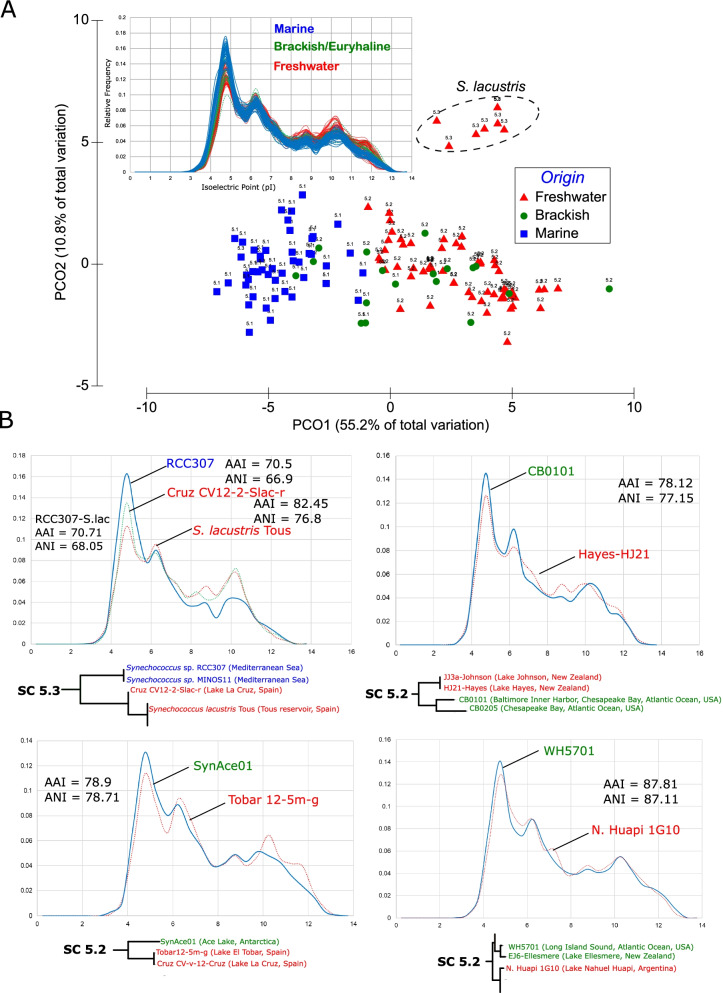
**A** Upper panel: Whole-proteome isoelectric points (pI, *x*-axis) versus relative frequency (*y*-axis) among different picocyanobacteria. Habitats are color coded accordingly. Lower panel: PCO plot based on a Bray-Curtis dissimilarity resemblance matrix obtained from the relative frequencies of 28 pI values (increments of 0.5 from 0 to 14). Each habitat is symbol and color coded accordingly. The SC to which each isolate belongs is also represented. **B** Whole-proteome pI comparison between close-phylogenetic neighbors. We provide a small inset of the phylogeny, AAI and ANI values. Dotted lines show the freshwater representative. Straight lines show the marine/brackish representative. A small inset of their phylogenetic affiliation is shown to highlight these pairs are the closest salt-adapted versus freshwater picocyanobacteria sequenced so far. Freshwater (red), brackish (green), and marine (blue) isolates are color coded accordingly

We next assessed the variation in isoelectric points (pI) in whole proteomes and constructed a principal coordinates analysis (PCO) based on a Bray-Curtis resemblance matrix for all 132 marine, brackish, and freshwater picocyanobacteria (Fig. 5A) building on a previous study which suggested that the changes at the level of protein amino acid composition and pI constitute a way to predict the preferred habitat of the different microorganisms [49]. The general phenomenon observed pointed towards a more acidic pI in all marine isolates compared to a more neutral and basic pI in freshwater isolates. Brackish, halotolerant, and estuarine picocyanobacteria showed either a pattern more related to marine isolates (e.g., WH5701, RS9917, RS9916, BS56D) or freshwater strains (e.g., NIES-98, NS01, or BSA11S/BSF8S).

These differences were also analyzed within sub-clades where we compared close-phylogenetic neighbors (with highest ANI and AAI values whenever possible) from different salinity types (Fig. 5B). In so doing, we aimed to reduce any taxonomic signal to a minimum. We performed four different comparisons: (i) RCC307 (Mediterranean Sea, marine) from SC 5.3 with *S. lacustris* and CV12-2-Slac-r (Tous reservoir and Lake La Cruz respectively, freshwater) from SC 5.2; (ii) Tobar-12-5-g (Lake El Tobar, freshwater) and SynAce (Ace Lake, brackish) both SC 5.2 representatives; (iii) CB0101 (Chesapeake Bay, brackish/estuarine) compared to Hayes-HJ21 (Lake Hayes, freshwater) both SC 5.2 representatives; (iv) WH5701 (Long Island Sound, brackish) compared to 1G10 (Nahuel Huapi, freshwater) also both SC 5.2 representatives. All these comparisons reiterated higher acidic pIs in the salt-adapted strains, while freshwater strains exhibited the highest neutral and basic pI peaks. Moreover, these comparisons also highlighted higher AAI values compared to ANI values in all cases, as previously noted [49].

### Habitat and picocyanobacterial sub-cluster specific metabolism in terms of gene/protein presence/absence

**Fig. 6:**
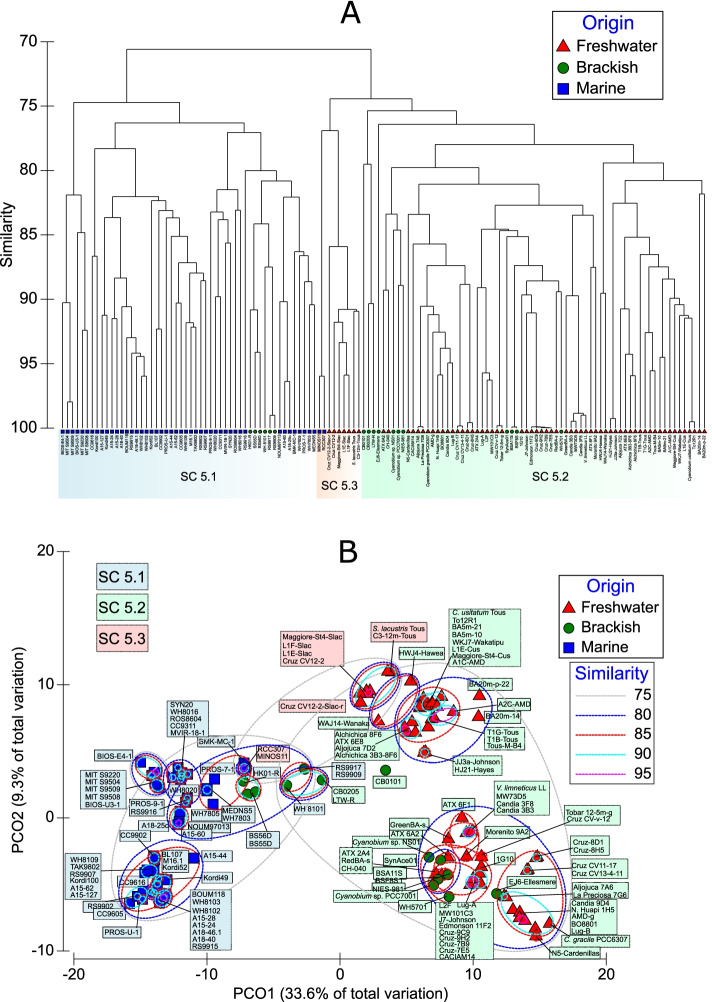
**A** Clustering and **B** PCO plots obtained from a resemblance matrix based on Cyanorak (CK) gene presence/absence (Kulczynski index). Both plots comprise all 132 picocyanobacteria labelled according to their habitat of origin and SCs. Overlayed clusters from 75 to 95% of similarity are shown in the PCO plot, determining the % of shared features between genomes

**Fig. 7:**
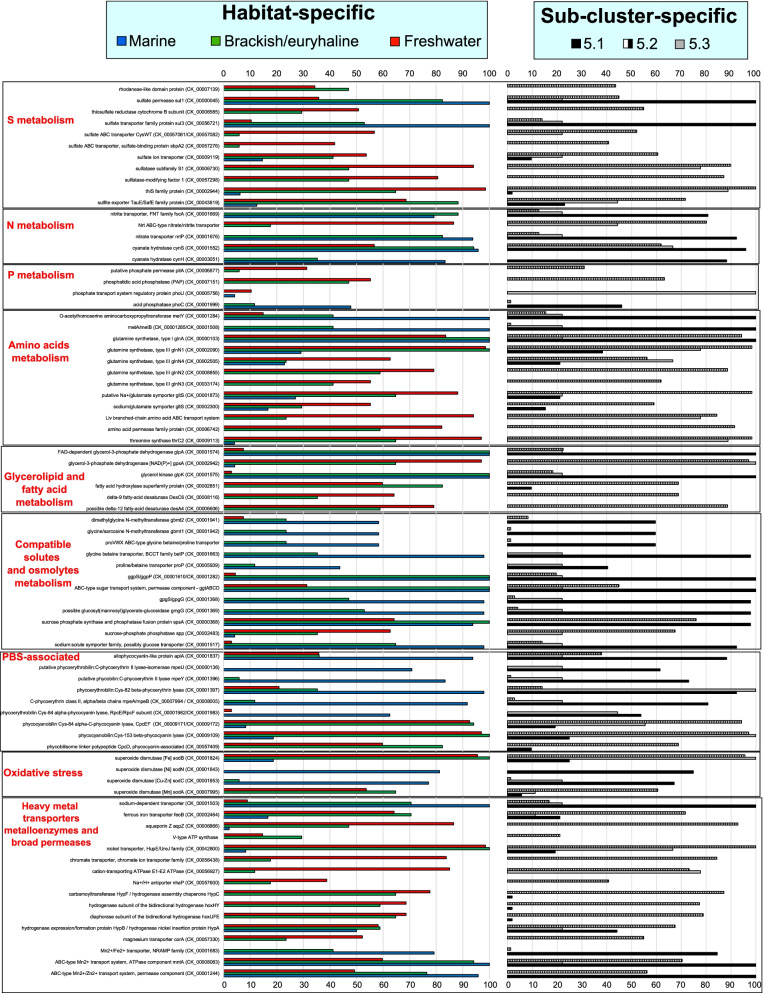
Picocyanobacterial habitat and sub-cluster (SC)-specific gene/protein presence/absence. Each habitat and SC are color coded accordingly. Presence/absence is based on total percentages of genomes that possess each gene/protein based on Cyanorak clusters (CK). We used 67 freshwater, 18 brackish, and 47 marine genomes sub-divided into SC 5.1 (51 genomes), SC 5.2 (72 genomes), and SC 5.3 (9 genomes)

To better understand what metabolic capacities differentiate salt-adapted and freshwater picocyanobacteria, we compared the presence/absence of various genes/proteins between habitats and SCs (Additional file 7: Table S2). Metabolic capacity used Cyanorak (CK) clusters [28] and compared 67 freshwater, 17 brackish, and 48 marine origin genomes sub-divided into 51 SC 5.1, 72 SC 5.2, and 9 SC 5.3 genomes. We verified CK annotations using KEGG, SEED, and EC numbers and assigned PSSMs based on CDD/SPARCLE. Based on all of the homology matches with the abovementioned CK database, we determined the presence/absence of each gene/protein variant. However, we must clarify that while a specific gene set may be absent in genomes obtained from one habitat type, it does not rule out that habitat type possessing a different gene set to do the same job, specifically with the number of hypothetical proteins that remain with unknown function. Moreover, this work deals with a new set of draft genomes that are not closed into a single contig. Hence, there could be a few genes/proteins present at the edges of broken contigs that are not detected. The 14,062 genes not present in Cyanorak clusters, mostly from the novel freshwater strains described here and representing the accessory/flexible genome (shell and cloud categories), were annotated with the last version of the NCBI nr database (Additional file 8: Additional dataset 4). A PCO and a clustering plot (Fig. 6) based on the presence/absence (Kulczynski index) of all genes derived from Additional file 7: Table S2 was also obtained. As depicted in Fig. 6, marine and freshwater picocyanobacteria grouped separately based on their gene presence/absence, with a clear separation between marine sub-clusters 5.1A and B as well as between freshwater strains. The latter comprised the majority of freshwater/brackish isolates from SC5.2 that grouped separately from the abovementioned smaller genomes of the cosmopolitan *S. lacustris* (SC5.3), *Cyanobium usitatum* (plus related *Cyanobium* spp. from SC5.2), and New Zealand Hawea/Wanaka strains from SC5.2. Subsequent habitat and sub-cluster-specific genes/proteins are shown in Additional files 9, 10, 11, 13, 14, 15, 16, 17, 19, and 21: Tables S2-S12, Fig. 7 and discussed below for each type of metabolism where there were ecologically significant similarities and differences:

#### Sulfur metabolism

Sulfur is one of the most abundant elements in seawater, not only in the form of sulfate but also within other forms like DMS and DMSP [50]. Conversely, it is much less abundant, in general, in freshwater systems [51] where it may be limiting for microbial life (e.g., in Lake Baikal [52];). Thus, we might expect a greater capacity for sulfur acquisition in freshwater isolates. Indeed, we found that the genomes of freshwater picocyanobacterial strains specifically harbored additional rhodaneses (CK_00007139), which catalyze the detoxification of cyanide and their subsequent conversion to thiocyanate, or (aryl) sulfatases such as sulfatase subfamily S1 (CK_00006730) involved in the transformation of phenol sulfate and water to phenol and sulfate, both of which were absent in marine strains (Fig. 7 and Additional file 9: Table S3). Also, of particular relevance here is the CysWT sulfate transporter, which is required for optimal growth of the freshwater strain *Synechococcus elongatus* [53] and was initially detected in some of the first freshwater picocyanobacterial MAGs from SC 5.3 [32]. This transporter has been mostly detected in freshwater and terrestrial cyanobacteria [54] compared to marine strains. Here, we detected the CysWT and CysPA sulfate transport system in over 50% (38/67) of the freshwater strains analyzed, mostly in members of SC 5.2 (albeit a few *S. lacustris* of SC 5.3 strains also possessed it), being completely absent from marine strains and present in only 1/17 brackish strains. Another sulfate ion transporter (CK_00009119) was present in 36/67 freshwater strains from SC 5.2 and 7/17 brackish strains, but interestingly, it was present in all strains from marine SC 5.3 and clades V, VIa/b from SC 5.1. On the other hand, sulfate permeases/transporters such as Sul1 (CK_00001149) or Sul3 (CK_00056721) were present in all marine strains and most brackish (15/17 and 9/17, for Sul1 and Sul3). However, only 26 and 8/67 freshwater strains harbored Sul1 and Sul3, respectively. Conversely, genes for assimilatory sulfate reduction were present in all the picocyanobacterial genomes analyzed (Additional file 9: Table S3), including phosphoadenylyl-sulfate reductase [thioredoxin] (CK_00001149), adenylylsulfate kinase (CK_00000454) and sulfite reductase (CK_00000887).

#### Nitrogen metabolism

Various studies have shown that nitrogen (particularly fixed forms such as ammonia and nitrate) is, together with P, the main limiting nutrient for phytoplankton growth [55]. This is consistent with the presence of ureases (123/132 possess the entire *ure* cluster), nitrate/nitrite reductases (120/132 harbor *nirA*, 119/132 possess *narM* and 118/132 contain *narB* genes), and ammonia permeases (*amt1* is present in all marine, 51/67 freshwater and 16/17 brackish strains; *amt2* is present in 68/132 genomes, particularly in 54/67 freshwater strains) in most but not all marine [19], freshwater, and brackish picocyanobacteria (Table S4). However, they all possess the global nitrogen regulator *NtcA* (CK_00000468) as well as the PII protein (*glnB* - CK_00000186). Interestingly, various freshwater (12/67) and brackish (3/17) isolates contain a second PII copy (*glnB2* - CK_00041583) (Additional file 10: Table S4). It seems possible that freshwater strains have evolved additional copies of this regulator together with additional glutamine synthetases (see amino acid section below) to cope with the variable nitrogen levels present in lakes of different trophic status.

On the other hand, while we found that the ability to degrade cyanate into ammonia and CO_2_ via cyanate hydratase was a common feature of marine and brackish representatives as previously noted [19, 56], this enzyme was present in only half (38/67) of the freshwater strains (Additional file 10: Table S4). It is possible that the prevalence of this bicarbonate-dependent enzyme in marine strains is correlated with the relative stability of ocean pH (generally ca. pH 8.2±0.3) [57], a feature that is much more variable in freshwater systems, e.g., from neutral to slightly alkaline in Lake Baikal [52] and Spanish reservoirs, meromictic Lake La Cruz (Spain) from which we have isolated different strains [58], or acid like in some French reservoirs [59]. Moreover, among all the compared planktonic picocyanobacteria, the only strain harboring a nitrogenase was the freshwater isolate *V. limneticus* spp. that acquired the *nif* operon via HGT [34]. Apart from this one exception, no other picocyanobacteria of all those analyzed here showed the ability to fix nitrogen. Finally, there were specific nitrate/nitrite transporters for marine and brackish strains such as the nitrate transporter *nrtP* (CK_00001676) and the *focA* nitrite transporter (CK_00001669) [60], which were absent in all freshwater strains. Conversely, freshwater isolates harbored the *nrt* ABC transporter, a well-defined nitrate/nitrite transporter in *S. elongatus* [61]. The exact reason why marine and freshwater microbes harbor different transporters for the same nutrients (either S or N) is unknown.

#### Phosphorus metabolism

Phosphorus (P) is another potentially limiting nutrient for picocyanobacterial growth across both marine and freshwater systems [62–64]. Many P metabolism genes were common to all picocyanobacteria regardless of their origin (Additional file 11: Table S5). Such features included the high-affinity PstS ABC phosphate transporter and PhnCDE phosphonate transport systems, the phosphate starvation-inducible protein (PhoH), or those involved in P storage and degradation of P polymers such as the *ppk* polyphosphate kinase (CK_00000383), *ppx* exopolyphosphatase (CK_00000620), *ppa* inorganic pyrophosphatase (CK_00000642), *rdgB* dITP/XTP pyrophosphatase (CK_00008108), or the nucleoside triphosphate pyrophosphohydrolase *mazG* (CK_00000805). Various alkaline phosphatases, involved in the degradation of organic P sources, were also common to virtually all picocyanobacteria and included the haloacid dehalogenase (HAD) family phosphatase (CK_00000983), a possible phosphatidic acid phosphatase (CK_00000999) and a DedA family protein (CK_00000302).

Despite the abovementioned shared P genomic features, we also identified some specific differences between strains (Additional file 11: Table S5). For example, the P regulatory protein PhoU (CK_00005756), which is known to play a role as a repressor in the PhoBR two-component system signal transduction process [65, 66], was unique to SC 5.3 isolates. This potentially may reflect the more variable P environments strains of this lineage occupy with the ability to repress P uptake when P concentrations are high and inhibit growth [67]. Furthermore, a gene annotated as a HAD phosphoserine phosphatase-like hydrolase, IB family protein (CK_00005504) was also only present in SC 5.3 strains (8/10), while a putative nucleotide phosphotransferase PPK2 (CK_00057252) was present in all SC5.3 strains and around a third of SC5.2 strains (20/57), but absent in the genomes of SC 5.1 isolates. Also noteworthy was the presence of two consecutive copies of the *phoB* gene (See Additional file 11: Table S5 and Additional file 12: Fig. S3) in 19 freshwater isolates from SC 5.2, with the majority of them closely related to the cosmopolitan strain *C. usitatum*. The sole marine strain harboring two copies of this gene in different parts of its genome was TAK9809. The presence of this extra copy of the phosphate regulon transcriptional regulatory protein in strains that are commonly found in multiple freshwater lakes (with varying P levels) may indicate a competitive advantage for these ecotypes in P fluctuating environments, albeit future experiments will be needed to clarify if both copies are functional.

Picocyanobacteria can also produce alkaline phosphatases under conditions of phosphate starvation [68]. Specific alkaline phosphatases were present in variable numbers across isolates from each habitat (Additional file 11: Table S5). In marine *Synechococcus* SC 5.1 strains it is known that some clades, e.g., clade III and WPC1 possess genomic adaptations to P-depleted environments, which include higher numbers of alkaline phosphatases [21, 63]. Also, some clade II marine isolates appear to have phosphite transport capacity [21, 69]. However, the latter trait appears largely absent in freshwater strains with a few exceptions (e.g., A2C-AMD, T1B-Tous, TIG-Tous, Tobar12-5m-g, and Tous-M-B4). We did though observe some specific signatures for various freshwater and brackish representatives. For instance, the phosphatidic acid phosphatase (PAP) (CK_00007151), putative purple acid phosphatase (CK_00007221) and a putative alkaline phosphatase (CK_00056881) were all absent from SC 5.1 and SC5.3 representatives but variably present in freshwater and brackish SC5.2 isolates (Additional file 11: Table S5). Interestingly, the PhoX alkaline phosphatase (CK_00009168) [68], detected in marine *Synechococcus*, was also present in virtually all of our freshwater (and brackish) genomes. In contrast, the inorganic phosphate permease PitA (CK_00006877), was present exclusively in around one-third (20/57) of SC 5.2 freshwater isolates (Additional file 11: Table S5). Curiously, this low affinity phosphate transporter was present in SC 5.2 isolates originating from lakes (Atexcac, La Cruz, Aljojuca) undergoing calcium carbonate precipitation phenomena where P concentration drastically drops below the limit of detection [58, 70, 71]. On the other hand, biomineralization experiments have shown that P can strongly affect the molecular composition of the *Synechococcus* cell surface, which in turn impacts CaCO_3_ precipitation [72]. Hence, a role for inorganic phosphate transporters in this process of carbon precipitation is conceivable.

#### Carbon fixation and photosynthesis

Being photoautotrophic, all genomes possessed a gene content consistent with their photosynthetic lifestyle, i.e., possessing photosystems I and II, NAD(P) H dehydrogenase, the cytochrome b6-f complex, ATP synthase, α-carboxysomes, type IA RuBisCOs (ribulose-1.5 biphosphate carboxylase-oxygenase) [47], and phycobilisomes (PBS) (Additional file 13: Table S6). We did note, however, some specific differences in some photosynthesis and light-harvesting genes. For example, the RuBisCO activase protein CbbX [73] was entirely absent from SC 5.3. We also found dissimilarites in the phycobilisome (PBS) complexes between strains isolated from different habitats (Additional file 13: Table S6). The chromatic adaptation ability of marine *Synechococcus* is well known [23–25, 74, 75] with strains possessing this capability able to modify the PUB-to-PEB ratio of the phycoerythrin II alpha chain to adapt to variable light conditions. As such, marine strains harbor C-phycoerythrin class II (*mpeA*/*mpeB*), associated rod linker polypeptides (*mpeCEGFH*) and phycoerythrobilin:phycoerythrin II lyases (*rpcE*/*rpcF*, *mpeZW*). However, all of these genes were absent from brackish (8) and freshwater (38) PE-rich strains that only possessed C-phycoerythrin class I (*cpeA*/*cpeB*) genes. Hence, possible chromatic adaptation of freshwater strains should be restricted to phycoerythrin I, a feature which remains to be tested in the laboratory, albeit previous studies have highlighted the discovery of a new type of pigmentation (IIb) in freshwater isolates with strains displaying additional subunits of phycoerythrin I [31, 35, 76].

Three types of phycocyanobilin lyases (*cpcT*, *cpcE*, *cpcF*) were also largely exclusive to freshwater and brackish strains, these being present in only four marine strains. Among the various PC-rich isolates we analyzed (29 freshwater and 9 brackish origin strains), we noted they harbored specific phycobilisome linker polypeptides, phycocyanin-associated, such as *cpcC1*/*C2*/*C3* (CK_00000012, CK_00057410, CK_00057409) that were absent from marine strains.

#### Amino acid biosynthesis

While the majority of amino acid biosynthetic pathways were present in all picocyanobacteria, we observed some differences in pathways that converged with N or S metabolism (Additional file 14: Table S7), which may be related to the abovementioned ecologically relevant differences between habitats. For instance, the enzymes homoserine O-succinyltransferase, O-succinylhomoserine(thiol)-lyase, and O-acetylhomoserine aminocarboxypropyltransferase, encoded by *metA*, *metB,* and *metY*, respectively, all of which are involved in both S and cysteine/methionine pathways, were virtually ubiquitous in marine strains (except *metB* that was lacking in marine SC 5.3). Conversely, *metA*/*metB* were absent in all freshwater isolates and only present in brackish/halotolerant strains from SC 5.1 (Additional file 14: Table S7). Thus, *metA*/*metB* appear to be restricted to SC 5.1. It is possible that marine strains use these enzymes as a substitute for the abovementioned freshwater sulfate/cysteine Cys transport system and alternative S pathways. On the other hand, cysteine synthase (CK_00008105), another enzyme participating in cysteine and S metabolism, does not appear in any of the marine/brackish members from SC 5.1, being only present in SCs 5.2 and 5.3.

Another key difference was observed in amino acids participating in N metabolic pathways such as glutamate and glutamine. All picocyanobacteria exhibited the ferredoxin-dependent glutamate synthase (*glsF*) yielding L-glutamate, but there were five different glutamine synthetases that convert glutamate and NH_3_ into glutamine, ADP, and phosphate, which were distributed differently between environments (Additional file 14: Table S7). Glutamine synthetase type I (*glnA*) was present in all picocyanobacteria except for freshwater SC5.3 strains. On the other hand, there were various differences in type III glutamine synthetases that are usually much larger (ca. 700 amino acids in length) compared to type I (450-470 amino acids in length). For instance, *glnN3* and *glnN2* were absent from all brackish and marine members of SC 5.1 and *glnN4* and *glnN1* were only present in clades I, V, and VI within SC 5.1. On the other hand, *glnN1* was present in all freshwater and brackish strains, while *glnN2*/*glnN3* were restricted to brackish members from SC 5.2. These additional glutamine synthetase subunits together with the extra *glnB* regulators may exemplify a much more variable repertoire of enzymes involved in N metabolic pathways in freshwater systems, where N levels strictly related to the trophic status of lakes, may be more variable compared to more homogeneous N-contents in off-shore marine ecosystems.

Finally, there was a set of ABC-type polar or branched chain amino acid transporters (*livKFGM*) that were widespread in freshwater and brackish isolates, but were absent in marine strains. Indeed, the presence of picocyanobacterial *livKFGM* genes was also higher in metagenomics datasets from lakes such as Lake Baikal (bathypelagic strata) compared to other marine meso- and bathypelagic systems [52].

#### Compatible solutes, osmolytes, salt related transporters, and ion channels

Genes particularly relevant to assessing the picocyanobacterial salinity divide are those encoding transporters and biosynthetic genes involved in osmolyte transport/biosynthesis and compatible solutes. If these were relevant, we would expect to see major differences between salt-adapted and freshwater microbes. In this respect, we found genes for the biosynthesis of amino acids and derivatives, well known for maintaining cellular osmotic balance, such as glycine betaine/L-proline ABC-transporters (*ProVWX*, *ProP* and *BetP*) to be particularly prevalent in salt-adapted picocyanobacteria, especially marine SC 5.1 strains, as previously noted [10, 19] (Additional files 15 and 16: Tables S8/S9). Similarly, genes involved in betaine biosynthesis/transport such as sarcosine N-methyltransferase (*gbmt1*), dimethylglycine N-methyltransferase (*gbmt2*), or the high-affinity choline uptake protein (BetT/betP, CK_00001663) were only present in marine and brackish representatives [10, 19].

Other sets of compatible solute biosynthetic pathways that were differentially spread among strains crossing the salinity divide were (i) those required for the biosynthesis of glucosylglycerol, comprising glucosylglycerol-phosphate synthase and glucosylglycerol 3-phosphatase as the main enzymes (encoded by *ggpS* and *ggpP*, respectively) with only two freshwater strains (CH-040 and ATX 6A2) possessing the *ggpSP* cluster, indicating potential growth in saline medium of these organisms, but present in all marine strains and virtually all brackish strains (16/17). A sodium:solute symporter family protein, possibly a glucose transporter (CK_00001517), was also present in all marine and 11/17 brackish strains, but present in only the 2/67 abovementioned freshwater strains CH-040 and ATX 6A2; (ii) the glucosylglycerate biosynthetic process (comprising glucosyl-3-phosphoglycerate phosphatase and glucosyl-3-phosphoglycerate synthase, encoded by *gpgP* and *gpgS*, respectively, and the glucosyl(mannosyl)glycerate-glucosidase encoded by *gmgG*). These were present in all marine strains and the majority of brackish (9/17 possess *gpgS/gpgP* and *gmgG*), but completely absent in freshwater isolates (Additional file 15: Table S8); (iii) the sucrose biosynthetic pathway (sucrose-phosphate synthase and phosphatase fusion protein encoded by *spsA*). Interestingly, the only marine isolates possessing the sucrose biosynthetic process comprising sucrose-phosphate phosphatase *spp* (CK_00002483) and sucrose-phosphate synthase *sps* (CK_00033172) genes were assigned to SC 5.3 (strains MINOS11 and RCC307). Conversely, *spp* was detected in 42/67 freshwater strains (but absent in all SC 5.3 freshwater isolates) and 6/17 brackish (all SC 5.2) isolates, while *sps* was present in 24/67 freshwater and 4/17 brackish strains. Additionally, *spsA* was found in 43/67 freshwater isolates but again absent in all SC 5.3 freshwater isolates (Additional file 15: Table S8); (iv) the maltose alpha-D-glucosyltransferase/alpha-amylase (CK_00001404), which was present in all marine and brackish representatives but only in 3/67 freshwater isolates (Additional file 15: Table S8). Similarly, cyclomaltodextrinases/neopullulanases (CK_00001576), which can protect against desiccation but also serve as fuel, producing energy by recycling the polymer pullulan [77], were present in most brackish (11/17) and marine (32/48) isolates, but practically absent from freshwater strains (4/67). Regarding the transport of compatible solutes, an ABC-type sugar transport system similar to the glucosylglycerol/trehalose/sucrose transporter Ggt (encoded by *ggtA, ggtB, ggtC, ggtD*) was ubiquitous in brackish and marine strains but only present in 21/67 freshwater isolates (and none from SC5.3) including strains from Lakes Aljojuca, Atexcac, Albano, La Cruz, La Preciosa, or Nahuel Huapi, all containing the entire *ggtABCD* gene cluster. However, the *ggtA* ATPase alone was found in an additional 12 freshwater strains (Additional file 15: Table S8).

We also determined the presence/absence of a broad range of other transporters (Additional file 16: Table S9), permeases and ion gated channels following previous predictions in marine picocyanobacteria [10, 19]. Differences observed included the distribution of small/large-conductance mechanosensitive ion channels (MscS, MscL), involved in the protection against hypo-osmotic shock, and in potassium transporters. For example, the divalent Anion:Na^+^ Symporter (DASS) or NhaS Na^+^ /H^+^ symporter were present in all picocyanobacteria. However, there were two large-conductance mechanosensitive channels, MscL, one of which (CK_00041811) was absent from freshwater strains but present in 21/48 marine and 3/17 brackish isolates. Conversely, the other MscL (CK_00002351) was more prevalent in freshwater (55/67) and brackish (8/17) strains than marine (17/48) representatives (Additional file 16: Table S9). We also detected variation in the presence/absence of 24 different MscS small mechanosensitive ion channels (Additional file 16: Table S9). Among these, we noted some that were particularly prevalent in freshwater environments, such as CK_00056919 and CK_00003081 (only absent in SC 5.3), CK_00008787 (51/67), and CK_00008021 (32/67). On the other hand, there were others that were absent from freshwater isolates and more widely distributed among marine and brackish strains, e.g., CK_0001534 (present in 4/17 brackish and 29/48 marine strains) or CK_00041767 (restricted to 36/48 marine SC 5.1 strains).

With regard to potassium transport, all picocyanobacteria possessed the DASS family, Kef efflux, Ktr uptake, and Trk exchanger systems. However, we noted a putative inward rectifier potassium channel (CK_00046459) was particularly prevalent in freshwater (45/67) and brackish (11/17) strains, all of them from SC 5.2, but absent from all marine isolates. Similarly, 43/67 freshwater, 7/17 brackish but only 8/48 marine strains possessed a putative potassium transporter CK_00002470. Such differences in potassium ion channels likely reflect differences in K^+^ concentrations between habitats and inside the cells of each particular microbe. Perhaps unsurprisingly then, genes responsible for the biosynthesis and transport of compatible solutes or involved in salt tolerance clearly separate marine, brackish, and freshwater picocyanobacterial isolates providing a molecular basis for understanding the salinity divide.

#### Glycerolipid and fatty acid metabolism

The differential adaptation to the salt environment also led us to consider differences in genes involved in lipid and fatty acid metabolism (Additional file 17: Table S10). All picocyanobacteria, regardless of their origin, possessed key fatty acid synthesis (FAS II) pathway genes including acetyl-CoA carboxylase (AccA-D), beta-ketoacyl-(acyl-carrier-protein) synthase II/III (KAS II/III), beta-ketoacyl-(acyl-carrier-protein) reductase (KR), beta-hydroxy-acyl-(acyl-carrier-protein) dehydratase (DH) and enoyl-(acyl-carrier-protein) reductase (ENR), as previously described in marine *Synechococcus* [78]. However, we observed several different glycerol-3-phosphate dehydrogenase variants in marine, brackish, and freshwater strains, with marine and brackish strains possessing a FAD-dependent version (*glpA*) and freshwater strains a NADPH-dependent one (*gpsA*), though curiously *gpsA* was also present in brackish isolates from SC5.2 which hence harbored both variants. Moreover, glycerol kinase (CK_00001575), involved in glycerolipid biosynthesis, was present in all brackish and marine isolates but only 2/48 freshwater isolates (CH-040 and ATX 6A2). Previous studies have noted the variable presence of fatty acid desaturases in marine and freshwater cluster 5 picocyanobacteria [78, 79]. Here, we detected various desaturases that were diversely spread among freshwater and brackish isolates but were essentially absent in marine strains. These included *desC* (CK_00056947), *desA4* (CK_00006606), *desC6* (CK_00008116), present in 17, 53 and 43/67 freshwater and 3, 10, and 6/17 brackish strains, respectively. Another desaturase, *desC4* (CK_00008117) was present in all brackish and virtually all freshwater (47/48) isolates, but was absent in 22/48 marine strains (all of them from clades II, III, WPC1, XX, and UC-A) (Table S10). Overall, the differences observed may hint at a different lipid composition in freshwater cluster 5 picocyanobacteria, a feature which will need to be determined experimentally to assess whether salt levels do indeed affect the lipidome of these organisms.

#### Anaerobic metabolism

Among the few anaerobic pathways found in picocyanobacteria (Additional file 7: Table S2), we detected both uptake (*hyp* genes) and bidirectional hydrogenases (*hox* genes) in the majority of freshwater and brackish picocyanobacteria, all of them from SC 5.2, similar to previous observations in freshwater filamentous cyanobacteria [80]. Conversely, while half (24/48) of marine strains harbored *hyp* genes, none of them showed bidirectional hydrogenases (*hox*). A feature encountered in a few freshwater and marine picocyanobacteria was the ability to ferment lactate (Additional file 7: Table S2). While FMN-dependent L-lactate dehydrogenase (CK_00004229) and D-lactate dehydrogenase (CK_00004230) variants were found in a few (3 and 6/48, respectively) marine strains, the L-lactate dehydrogenase (CK_00052344) variant was exclusive to 15/67 freshwater strains from SC 5.2. Noteworthy here is that a PE-rich *Synechococcus* isolated from mesopelagic euxinic waters in the aphotic zone (750 m) of the Black Sea [31] and two coastal isolates from the same habitat [30], all possess D-lactate dehydrogenase and therefore the ability to ferment lactate, potentially explaining their presence and growth in the dark mesopelagic meromictic Black Sea (1000 cells mL^−1^ at 750 m) [31]. Fermentation together with mixotrophy can provide maintenance metabolic capacity under dim light or dark conditions for a long time in deep anaerobic environments or until physical (e.g., turbulence) or biological processes (e.g., buoyant density changes) return cells to the photic layer, as was demonstrated for lacustrine filamentous cyanobacteria [81, 82].

#### Vitamin biosynthesis

Genes required for the biosynthesis of folate such as dihydrofolate reductase (CK_00040005 and CK_00057273) were found in 21/67 freshwater and 2/17 brackish strains, all of them from SC 5.2, but were absent from marine isolates (Additional file 7: Table S2). Among other enzymes involved in the biosynthesis of various cofactors/porphyrin, vitamin B12, and chlorophylls, the decarboxylating precorrin-6Y C5,15-methyltransferase (*cbiT* - CK_00002935), which participates in the aerobic (late cobalt insertion) adenosylcobalamin biosynthesis pathway, was only present in SCs 5.2 and 5.3 and hence absent from SC 5.1 genomes. Conversely, the remaining precorrin genes such as *cobH* (CK_00000320) (missing in only 5/132 genomes), cobalamin synthase *cobS* (CK_00000250) (missing in only 2/132 genomes), pseudocobalamin biosynthesis protein CobW (*cobW* - CK_00000869), *cbi* genes, cobalt chetalase (*cobN* - CK_00008103), and cob(I)alamin adenosyltransferase (*cobO1*/*cobO2*) involved in the biosynthesis of pseudocobalamin, which has been demonstrated for SC 5.1 *Synechococcus* [83], were present in virtually all genomes (Additional file 7: Table S2). Noteworthy, we did not detect *bluB*, *CbiZ*, and anaerobic vitamin B12 biosynthetic genes (*BzaABCDE*) in our isolates. Another enzyme that was specific to SC 5.3 and 5.2 isolates was threonine synthase (CK_00009113), involved in vitamin B6, glycine, and serine/threonine metabolism, which was compeletely absent from marine members of SC 5.1.

#### Oxidative stress

In marine SC 5.1 *Synechococcus*, superoxide dimutases that are critical for dealing with reactive oxygen species (ROS) are primarily nickel [84, 85] and copper/zinc [86] variants of the enzyme, although a few strains exhibit manganese or iron versions [19]. However, a detailed phylogenetic analysis of our new freshwater and brackish picocyanobacteria showed a clear bias towards iron and manganese superoxide dimutases, with the nickel variant being absent from all freshwater genomes (Additional file 18: Fig. S4 and Additional file 19: Table S11). This is consistent both with a larger survey including various filamentous and heterocystous cyanobacteria from both aquatic and terrestrial habitats [54], and with potentially widespread iron limitation in high nutrient low chlorophyll oceanic regions affecting phytoplankton growth [87–89], which potentially contrasts with freshwater systems, e.g., lakes like Lake La Cruz (from which we obtained several of our freshwater isolates) which exhibit relatively high iron concentrations [58, 90].

Interestingly, the *katG* catalase/peroxidase (CK_00001897) was present in 51/67 freshwater, 13/17 brackish but only 19/48 marine strains. However, many other enzymes involved in ROS protection were globally distributed in picocyanobacteria (Additional file 19: Table S11), including thiol peroxidase Bcp-type (EC 1.11.1.15), glutathione peroxidase (CK_00000308), rubredoxin (CK_00000269), monothiol glutaredoxin (CK_00000743), glutaredoxin 3 (CK_00000445), thioredoxin 1 (x-type) (only absent in marine CRD1 clade), *trxA* (CK_00008028), NTR system (*trxB*), and the ferredoxin-thioredoxin reductase.

#### Metal-related enzymes/transporters and other permeases

Given the data obtained for superoxide dismutases, we wondered if other metalloenzyme and heavy metal transporters might also show a bias towards a specific habitat type. Thus, ferrous iron transporter proteins such as *feoB* were mostly present in freshwater (43/67) and brackish (12/17) picocyanobacteria, being present in only 8/48 marine strains mostly from the CRD1 clade. On the other hand, ferrochelatase (CK_00000664) and the Fur regulator were present in all isolates regardless of origin (Additional file 16: Table S9). Similarly, the Mnt ABC-type Mn^2+^ transporter, membrane component (CK_00000080) was present in all marine strains, 16/17 brackish strains but only 40/67 freshwater strains. In contrast, a potential zinc transporter, ZupT, initially detected in freshwater *S. lacustris* MAGs [32], appears to be restricted to *S. lacustris* and *Cyanobium usitatum* isolates [34] both isolate types being obtained from the ultraoligotrophic Lake Baikal and oligotrophic Tous reservoir. Furthermore, the magnesium transporter *corA* (CK_00057330) was unique to freshwater and brackish strains from SC 5.2, but absent in marine isolates. Finally, a Mn^2+^ and Fe^2+^ NRAMP-type transporter (CK_00001683) was found in 38/48 marine strains (missing in SC 5.3) but was absent in all freshwater strains. Hence, it appears that in some cases marine and freshwater strains have evolved slightly different transporters for oligoelements such as Zn, Mn, or Fe.

Regarding other transporters, another obvious distinction between habitat types was the aquaporin Z water channel protein (CK_00006866), which was detected in virtually all freshwater (except for SC 5.3) and brackish picocyanobacteria from SC 5.2 but was essentially absent in marine strains being only present in BMK-MC-1 (Additional file 16: Table S9). Also noteworthy was the presence of the vacuolar V-type ATP synthase in freshwater (9/67) and brackish strains (5/17) being exclusively found in SC 5.2, while again there was no evidence of this ATPase type in marine strains (Additional file 16: Table S9).

#### Phage-related systems and mobile genetic elements

Alongside the general reduction in genome size in marine picocyanobacteria, we also observed a generally lower abundance of transposases and other mobile genetic elements in marine strains (Additional file 20: Fig. S5A and Additional file 21: Table S12) compared to their freshwater and brackish counterparts. In terms of the total number of transposases, freshwater and brackish (average values of 20.3 and 27.5, respectively) contained ca. ten times more genes compared to marine isolates (average of 1.89 transposases/genome). We observed >40 in most brackish representatives such as BSA11S/BSF8S (Black Sea), CB0101 (Chesapeake Bay), WH5701 (Long Island), NS01 (North Sea), or NIES-981 (Additional file 20: Fig. S5A). Various freshwater isolates such as *C. gracile* or those from New Zealand, Lake La Cruz, or Lake Baikal possessed >20 transposases per genome, but only three marine strains possessed >10 (ROS8604, BMK-MC-1 and A15-44) with the majority of marine isolates devoid of them (Additional file 20: Fig. S5A and Additional file 21: Table S12). On the other hand, phage integrases were distributed in the majority of strains regardless of their isolation environment (Additional file 20: Fig. S5B and Additional file 21: Table S12).

The only picocyanobacterium harboring a conjugative transposon cluster (*tra* genes) was a freshwater strain obtained from Lake Hawea (HWJ4-Hawea), a genome that, curiously, was among the smallest of all compared in this work (2.3 Mb). With regard to phage defense systems and phage-related proteins, we encountered only a few freshwater picocyanobacteria with clustered regularly interspaced short palindromic repeat (CRISPR-Cas) systems (*S. lacustris* Tous, ATX-2A4, *Vulcanococcus limneticus,* MW73D5, Candia 3F8 isolates), most of which encoded the Cas1, Cas2, Cas3, CT1974 (Cse3), CT1976 (Cas5) proteins (Table S12), or having a putative novel type III system as previously described in the *S. lacustris* Tous isolate [33].

#### Circadian genes

All picocyanobacteria analyzed here possess the two-component sensor histidine kinase SasA (CK_00000993), circadian phase modifier cpmA (CK_00000726), and circadian clock proteins KaiABC (Additional file 7: Table S2). However, the circadian input kinase was missing in some freshwater and brackish representatives. Finally, the circadian period extender Pex enzyme (CK_00001690) was more prevalent in marine picocyanobacteria, and specifically in SCs 5.3 and 5.1, being present only in freshwater isolates from the *S. lacustris* clade but absent entirely in SC 5.2.

## Conclusions

This work should be considered an ecogenomics starting point given that many more ecological, physiological, biochemical, and “omics” (transcriptomics, proteomics, lipidomics) studies will be required to completely disentangle the picocyanobacterial salinity divide. Nonetheless, the comparative genomics we report here is beginning to more precisely define the various genomic features that might differentiate marine, brackish, and freshwater picocyanobacteria. Thus:

1. There is a clear genome reduction in marine picocyanobacteria (SC 5.1 and SC 5.3) as observed in:
  ▪ A smaller average genome size and lower %GC content.
  ▪ A higher percentage of core and soft core genes compared to freshwater isolates.
  ▪ A lower potential metabolic capacity compared to the larger freshwater genomes including the absence of some specific pathways for sulfur metabolism.
  ▪ A common lack of mobile elements such as transposases.

(2) The shared and flexible genome clearly varies between SCs and habitats:
  ▪ The strict core stabilizes around 351 genes (10% of the total) between all habitats and SCs, while the soft core stabilizes around 1204 genes (35% of the total).
  ▪ The picocyanobacterial pangenome is much larger than 35,000 genes.

(3) Marine picocyanobacteria exhibit higher acidic and less basic isoelectric point patterns in their proteome. This adaptation is conserved between marine SCs 5.1 and 5.3, and freshwater SCs 5.2 and 5.3, but is also habitat-conserved when comparing close-phylogenetic neighbors.

(4) Picocyanobacteria possess specific habitat and SC-specific metabolic capacity:
  ▪ Marine strains possess the capacity for salt tolerance (e.g., the biosynthesis of glycine betaine, or the presence of sodium transporters), accentuate the use of zinc/copper/nickel superoxide dismutases, and virtually all hydrolyse cyanate as a N source.
  ▪ Freshwater strains retain some genes involved in anaerobic metabolism (hydrogenases, nitrogenase, fermentative pathways), which may reflect the wider niche diversity of freshwater isolates, a preference for iron/manganese metalloenzymes, branched chain, and polar amino acid metabolism, a more versatile repertoire of S metabolic genes (sulfate/thiosulfate transporters, rhodanese or arylsulfatases), and ca. 10 times more mobile elements such as transposases.
  ▪ Brackish isolates contain features of both marine and freshwater strains, highlighting the dynamic estuarine or brackish environments these strains inhabit, where marine and freshwater systems interconnect.

Future genomic studies should aim to completely close these freshwater genomes, as has recently been done for most marine isolates [21], which will facilitate absolute gene presence/absence work, and more easily allow studies of gene synteny, genomic island predition, adaptation, etc. Physiologically, a thorough assessment of growth across a range of salt concentrations is required for the isolates we report here. Ultimately, there is also a requirement to obtain axenic cultures of several strains, especially to assess specific nutrient and growth requirements. Finally, given the genomic diversity seen within freshwater SC 5.2 isolates, it is possible that specific lineages occupy distinct ecological niches likely dictated by the specific temperature, light, and nutrient characteristics of specific freshwater environments, or even other ecological factors, information which is clearly lagging behind that known for marine SC 5.1 clades [8, 42, 91].

## Methods

### Isolation campaign of freshwater picocyanobacteria

The isolation strategy for the freshwater strains obtained here was based on previously described approaches [33–35, 92]. All isolates were ultimately grown in either normal or twofold diluted BG11 medium. In some cases (Spanish isolates), initial culture development required the use of cycloheximide (1 mg/mL) to remove eukaryotic algae, and BG11 supplemented with vitamin B12 (0.015 μg/ mL). A dilution to extinction approach was applied to obtain some isolates (Spanish) whereas others [92] were obtained through filtration and flow cytometric single-cell sorting (InFlux V-GS flow cytometer, Becton Dickinson Inc) [93]. Strains originating from New Zealand were isolated using MLA medium [94]. Although all our obtained picocyanobacterial cultures were unialgal they were not axenic. However, in all cultures picocyanobacteria represented >75% of all cells as monitored by flow cytometry, microscopy and as depicted from the total number of sequences (see below). All our freshwater isolates are available from the MEG-Verbania [92] and University of Valencia cyanobacterial culture collections.

### DNA extraction and sequencing, read assembly and contig annotation

DNA from the newly described freshwater strains was extracted using two different methods: either the EZNA soil DNA extraction kit (Omega Bio-Tek) [95] or using a CTAB-lysis buffer followed by phenol-chloroform-isoamyl alcohol extraction [96], the latter providing higher recovery amounts.

Genomic DNA from most strains was sequenced using an Illumina NovaSeq PE150 (Novogene, UK/Hong Kong). Approximately, 1 Gb sequence data was obtained per sample. However, the New Zealand strains were sequenced using an Illumina MiSeq PE250 and Illumina TruSeq Nano DNA 550 bp UDI library preparation technology (University of Otago, New Zealand), again yielding ~1 Gb sequence data per sample. Samples were then individually cleaned with Trimmomatic v0.39 [97], assembled with SPAdes [98] following --careful, --only-assembler, -k 57,67,77,87,97,107,117,127, -t 48, -m 250 parameters. Finally, contigs were manually inspected using the following annotation pipeline: ORF prediction was assessed using Prodigal [99], then the functional annotation and taxonomy of each CDS and contig was assessed with Diamond BLAST [100] against the nr database. Proteins were annotated with the latest NCBI nr, KEGG [101], SEED [102], COG [103], and TIGFRAMs [104] databases to provide the most updated and robust nomenclature and taxonomy. tRNAs and rRNAs were detected with tRNAscan-SE 2.0.5 [105] and ssu-align [106], respectively. Based on these taxonomic and annotation results, we manually separated cyanobacterial contigs from heterotrophic bacteria. Metabat2 [107], checkM [108], and GTDB [109] were also used whenever necessary to separate and bin together cyanobacterial contigs, removing any remaining contamination from other bacteria.

### Phylogenomics and individual phylogenetic trees

Phylogenomics used a 365-protein concatenated tree obtained via the PhyloPhlAn3 tool [110] using the following parameters: -d phylophlan -t a --diversity high --accurate -f configs/supermatrix_aa.cfg. We exclusively used culture-derived marine (either complete or draft genomes), brackish, and freshwater picocyanobacteria from SCs 5.1, 5.2, and 5.3, but also 7 *Prochlorococcus* and 7 *Ca*. Synechococcus spongiarum and rooted the phylogeny using *S. elongatus* [111], Yellowstone, and PCC clade strains (16 genomes in total).

An individual phylogeny for superoxide dismutases was obtained by aligning individual proteins with MAFFT [112] and then using the IQ-TREE tool [113] with the following parameters -bb 1000 -nt AUTO -alrt 1000 to determine the best model for each protein type.

### Pangenomic approach between habitat and sub-clusters

To determine the percentage of shared and flexible genes between different picocyanobacteria, we analyzed through reciprocal gene homology identification [48] the pangenome of all SCs 5.1, 5.2, and 5.3 representatives. We only used closed or draft genomes derived from cultures to minimize the bias from SAGs or MAGs that are often incomplete and may miss important parts of the core or flexible genome. We obtained the percentage of genes that belonged to the core and soft core as well as the flexible genome (shell/cloud) using previously described approaches [114, 115]. Briefly, and as previously defined [48], the strict core comprised genes present in all our compared genomes, while the soft core comprised genes present in 95% of the compared picocyanobacterial genomes. The shell category comprised moderately conserved genes present in <90% genomes from all habitat groups (marine, freshwater, or brackish). Finally, cloud genes (strain-specific) comprised those rare genes present in only one or two genomes.

### Isoelectric points of different picocyanobacteria, whole-proteome comparison

The whole-proteome and the pIs of individual picocyanobacterial proteins were obtained using PEPSTATS, from the EMBOSS package [116]. To assess differences in the whole proteomes of marine, freshwater, and brackish picocyanobacteria, we constructed a PCO plot based on a Bray-Curtis resemblance matrix, which was previously transformed (square root) and obtained from the relative frequencies for each pI (0–14) with an increment of 0.5. Transformation of the data (relative frequencies), produced a Bray-Curtis similarity matrix and a PCO plot using the PRIMER6 tool [117].

### Marine, brackish, and freshwater gene/protein presence/absence and metabolic comparisons

To detect gene/protein presence/absence, we annotated all genomes with Prokka v 1.14 [118] using the latest release (May 2021) of the Cyanorak database [28] in a customized protein database. The 14,062 non-clustered Cyanorak genes were annotated with the latest version of the NCBI nr database (Additional file 8: Additional dataset 4). All clustered CK genes were double checked for gene/protein presence/absence using various databases as follows: KEGG [101], SEED [102], COG [103], TIGRFRAMs [104], and BLAST [119] versus the NCBI nr database. Additionally, we assigned PSSM values for each protein type following CDD/SPARCLE [120] searches. These BLASTp searches were performed with Diamond [100], obtaining top hits with at least 30% amino acid identity and >50% query sequence coverage. To assess metabolic differences between marine, freshwater, and brackish picocyanobacteria, we constructed a Kulczynski resemblance matrix based on presence/absence gene values. Starting from the obtained triangular matrix, we then performed a clustering and PCO analysis where genomes were distributed accordingly and multiple gene correlations were also shown and plotted.

### Publicly available picocyanobacterial strains derived from cultures

All marine picocyanobacterial genomes used in this work were obtained from Cyanorak [28] as described recently [21]. We also used previously sequenced freshwater and brackish isolates [30, 31, 33–35, 92] together with our newly sequenced strains (see also [47]). Details of all the genomes used in this work and their genomic features, origin, and references are provided in Additional file 1: Table S1. All genomes were deposited in the NCBI-Genbank database under Bioproject number PRJNA718564, Biosample numbers SAMN18541576-SAMN18541633 and Genbank accession numbers JAGQDB000000000-JAGQAY000000000.

## Supplementary Information

**Additional file 1: Table S1.** Main genomic features of marine, brackish and freswhater culture-derived picocyanobacteria. An asterisk in the origin column indicates strains where euryhaline physiology is known.

**Additional file 2: Fig. S1.** Average Nucleotide Identity (ANI) matrix between all 132 compared picocyanobacteria from SCs 5.1, 5.2 and 5.3.

**Additional file 3: Additional Dataset 1.** Average and standard deviation genome size, median intergenic spacers, coding density and %GC of all culture-derived picocyanobacteria. Single pair ANOVA tests for each origin and sub-cluster and for different genomic features.

**Additional file 4: Fig. S2**. A) Cross-comparison of strict core, soft core, shell and cloud in all 132 picocyanobacteria from all habitats and SCs. B) Plots estimating the core genome (n° of genes) and pangenome (n° of genes) of all three SCs. C) Functionality of the meta-pangenome of picocyanobacteria assessed by SEED/KEGG. Each gene category is color coded for the shared (strict core, soft core) and flexible (shell and cloud) genome.

**Additional file 5: Additional Dataset 2.** Pangenomic analysis between all picocyanobacterial isolates from SCs 5.1, 5.2 and 5.3. The total number of genes for each category (strict core, soft core, shell and cloud) and isolate are specified.

**Additional file 6: Additional Dataset 3.** 5.1-5.2-5.3 meta-pangenome. Core, strict-core, shell and cloud annotated genes with Cyanorak clusters (CK) and SEED.

**Additional file 7: Table S2.** Gene/protein presence/absence between all 132 compared culture derived marine, brackish and freshwater picocyanobacteria. Annotation assessed by Cyanorak CK clusters.

**Additional file 8: Additional Dataset 4.** Non-clustered CK genes from all 132 analyzed picocyanobacteria. BLASTP results obtained with the closest taxon from the NCBI nr database.

**Additional file 9: Table S3.** S metabolism. Gene/protein presence/absence between all 132 compared culture derived marine, brackish and freshwater picocyanobacteria. Annotation assessed by Cyanorak CK clusters. CDD was used to retrieve the PSSM-ids from best specific/non-specific hits covering >50 % of the protein.

**Additional file 10: Table S4.** N metabolism. Gene/protein presence/absence between all 132 compared culture derived marine, brackish and freshwater picocyanobacteria. Annotation assessed by Cyanorak CK clusters. CDD was used to retrieve the PSSM-ids from best specific/non-specific hits covering >50 % of the protein.

**Additional file 11: Table S5.** P metabolism. Gene/protein presence/absence between all 132 compared culture derived marine, brackish and freshwater picocyanobacteria. Annotation assessed by Cyanorak CK clusters. CDD was used to retrieve the PSSM-ids from best specific/non-specific hits covering >50 % of the protein.

**Additional file 12: Fig. S3.** Genomic context of the *phoBR* two-component system in different marine, brackish and freshwater cluster 5 picocyanobacteria. Each subunit is color coded accordingly. The right panel shows a phylogenomic tree with all those freshwater strains (colored red) possessing two copies of the *phoB* gene.

**Additional file 13: Table S6.** C fixation/photosynthesis. Gene/protein presence/absence between all 132 compared culture derived marine, brackish and freshwater picocyanobacteria. Annotation assessed by Cyanorak CK clusters. CDD was used to retrieve the PSSM-ids from best specific/non-specific hits covering >50 % of the protein.

**Additional file 14: Table S7.** Amino acid metabolism. Gene/protein presence/absence between all 132 compared culture derived marine, brackish and freshwater picocyanobacteria. Annotation assessed by Cyanorak CK clusters. CDD was used to retrieve the PSSM-ids from best specific/non-specific hits covering >50 % of the protein.

**Additional file 15: Table S8.** Compatible solutes and osmolytes. Gene/protein presence/absence between all 132 compared culture derived marine, brackish and freshwater picocyanobacteria. Annotation assessed by Cyanorak CK clusters. CDD was used to retrieve the PSSM-ids from best specific/non-specific hits covering >50 % of the protein.

**Additional file 16: Table S9.** Broad transporters, permeases, channels and uptake systems. Gene/protein presence/absence between all 132 compared culture derived marine, brackish and freshwater picocyanobacteria. Annotation assessed by Cyanorak CK clusters. CDD was used to retrieve the PSSM-ids from best specific/non-specific hits covering >50 % of the protein.

**Additional file 17: Table S10.** Glycerolipid/Fatty acid metabolism. Gene/protein presence/absence between all 132 compared culture derived marine, brackish and freshwater picocyanobacteria. Annotation assessed by Cyanorak CK clusters. CDD was used to retrieve the PSSM-ids from best specific/non-specific hits covering >50 % of the protein.

**Additional file 18: Fig. S4.** Phylogenetic analysis of picocyanobacterial Ni/Cu/Zn/Fe/Mn superoxide dismutases. The Ni-type maturation protease from marine strains was used to root the tree. Bootstrap values >75 are shown and the habitat of each picocyanobacterial enzyme is color coded.

**Additional file 19: Table S11.** Reactive oxygen species (ROS). Gene/protein presence/absence between all 132 compared culture derived marine, brackish and freshwater picocyanobacteria. Annotation assessed by Cyanorak CK clusters. CDD was used to retrieve the PSSM-ids from best specific/non-specific hits covering >50 % of the protein.

**Additional file 20: Fig. S5.** Total number (Y axis) of A) transposases and B) integrases found in freshwater, brackish and marine picocyanobacteria.

**Additional file 21: Table S12.** Mobile genetic elements. Gene/protein presence/absence between all 132 compared culture derived marine, brackish and freshwater picocyanobacteria. Annotation assessed by Cyanorak CK clusters. CDD was used to retrieve the PSSM-ids from best specific/non-specific hits covering >50 % of the protein.

## Data Availability

All data generated or analyzed during this study are included in this published article, its supplementary information files and publicly available repositories. All data derived from this work is publicly available in NCBI-Genbank databases.

## Abbreviations

SC: Sub-cluster
pI: Isoelectric point
MAG: Metagenome assembled genome
ANI: Average nucleotide identity
AAI: Average amino acid identity
SAG: Single-cell amplified genome
CK: Cyanorak
ROS: Reactive oxygen species
PCO: Principal coordinates analysis
PAP: Phosphatidic acid phosphatase
RuBisCO: Ribulose-1.5 biphosphate carboxylase-oxygenase
PBS: Phycobilisomes
FAS: Fatty acid synthesis
AccA-D: Acetyl-CoA carboxylase
KAS II/III: Beta-ketoacyl-(acyl-carrier-protein) synthase II/III
KR: Beta-ketoacyl-(acyl-carrier-protein) reductase
DH: Beta-hydroxy-acyl-(acyl-carrier-protein) dehydratase
ENR: Enoyl-(acyl-carrier-protein) reductase
CRISPR: Clustered regularly interspaced short palindromic repeats
