# Supplementary figures and images for: Elucidating the picocyanobacteria salinity divide through ecogenomics of new freshwater isolates
Source: BMC Biol. 2022 Aug 8;20:175. doi: 10.1186/s12915-022-01379-z (PMC9361551; doi:10.1186/s12915-022-01379-z)

# Average Nucleotide Identity %

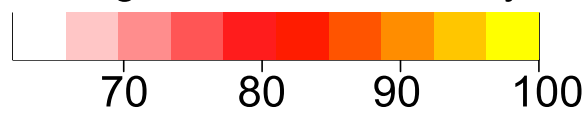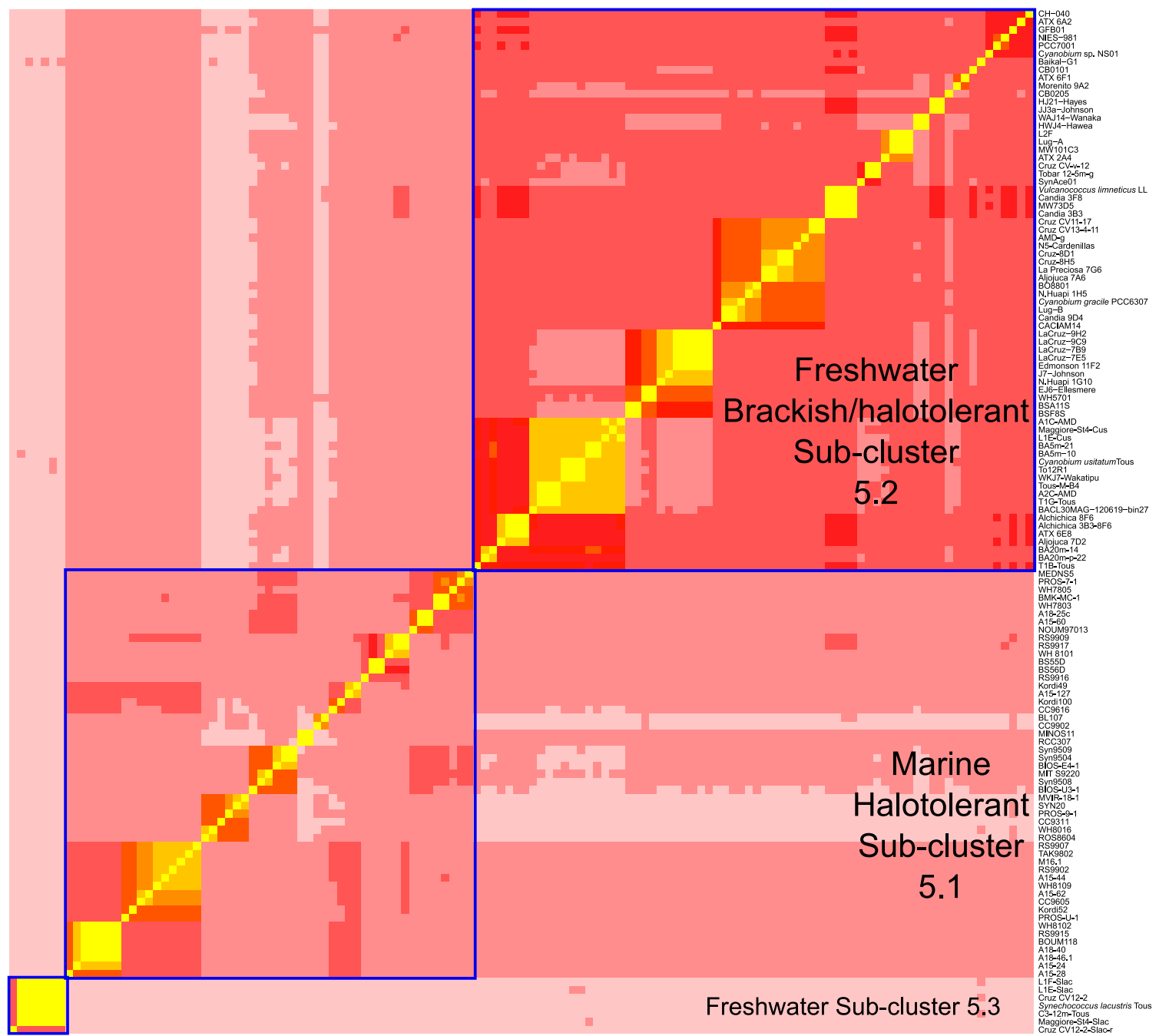

Supplement: Supplementary file 2 — Additional file 2: Fig. S1. Average Nucleotide Identity (ANI) matrix between all 132 compared picocyanobacteria from SCs 5.1, 5.2 and 5.3. [file 12915_2022_1379_MOESM2_ESM.pdf]

A

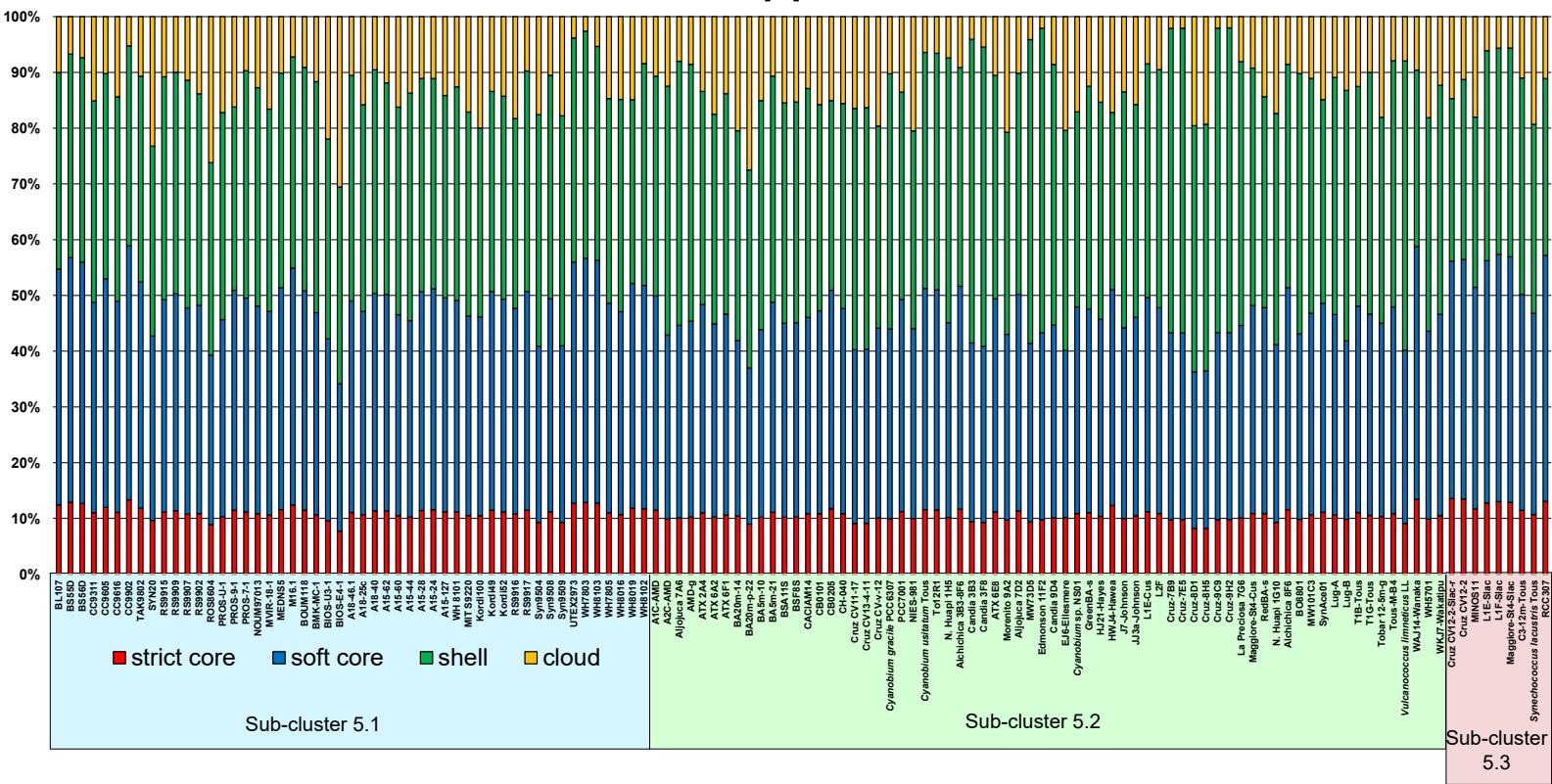

B

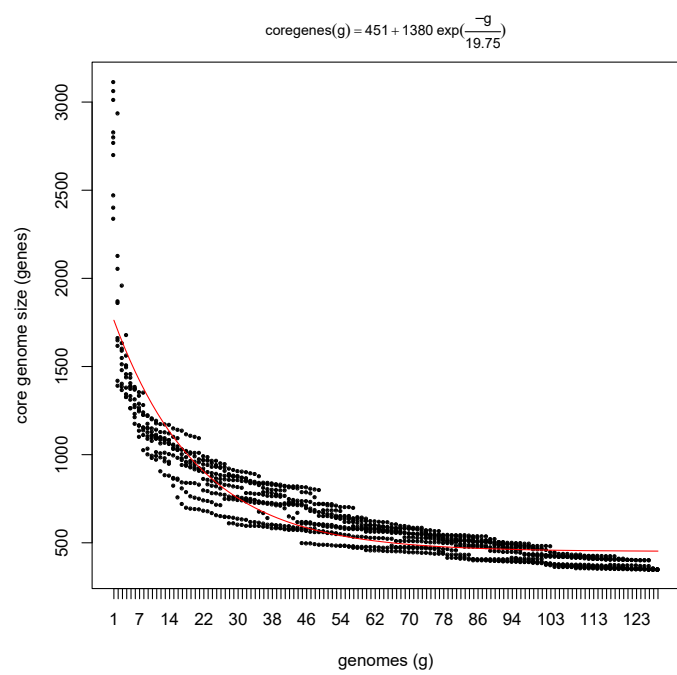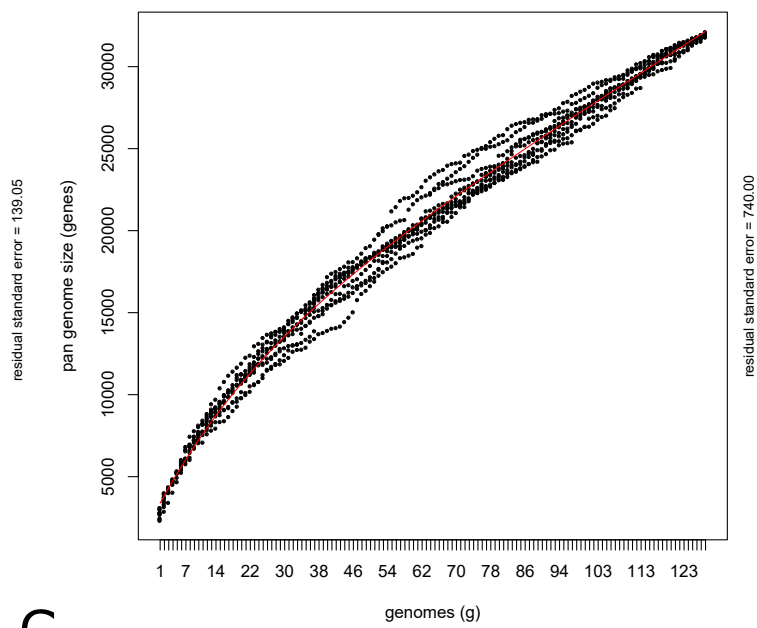

C

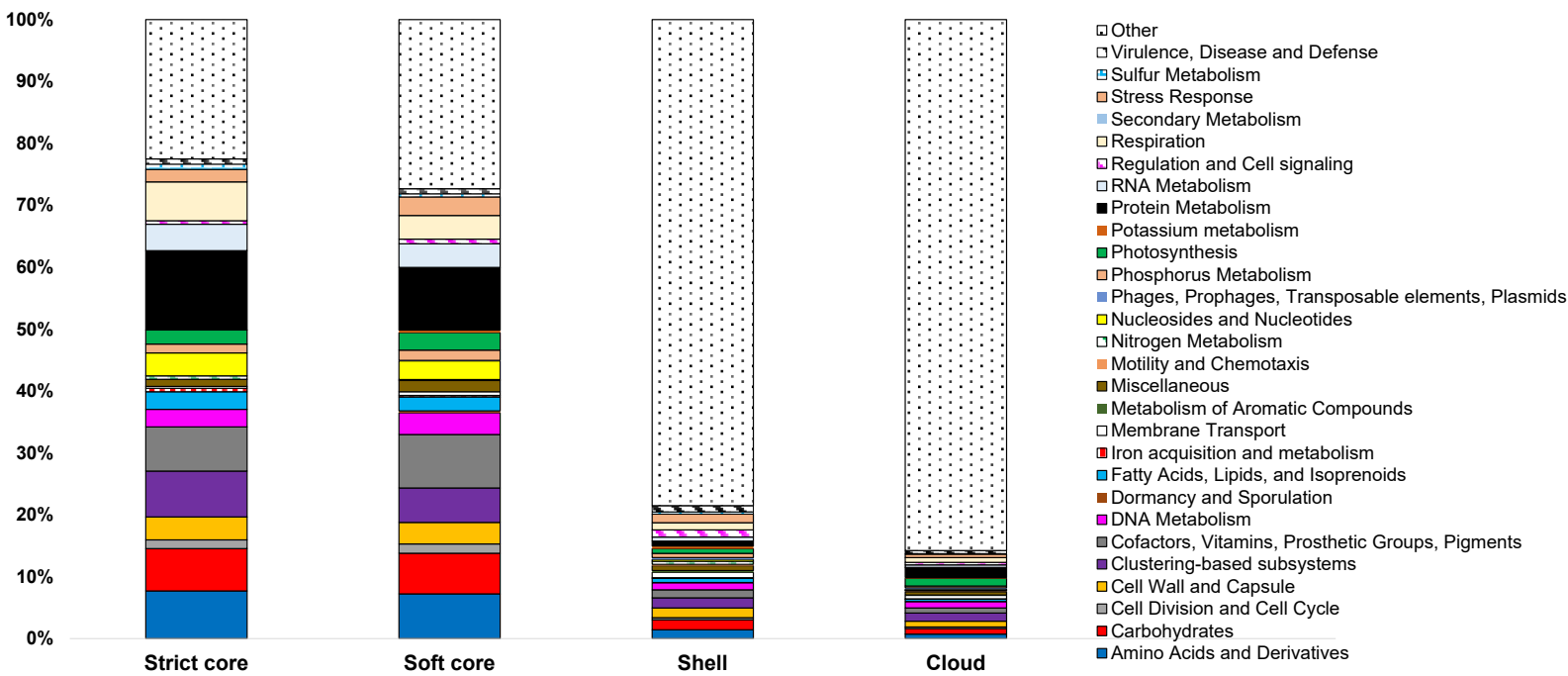

Supplement: Supplementary file 4 — Additional file 4: Fig. S2. A) Cross-comparison of strict core, soft core, shell and cloud in all 132 picocyanobacteria from all habitats and SCs. B) Plots estimating the core genome (n° of genes) and pangenome (n° of genes) of all three SCs. C) Functionality of the meta-pangenome of picocyanobacteria assessed by SEED/KEGG. Each gene category is color coded for the shared (strict core, soft core) and flexible (shell and cloud) genome. [file 12915_2022_1379_MOESM4_ESM.pdf]

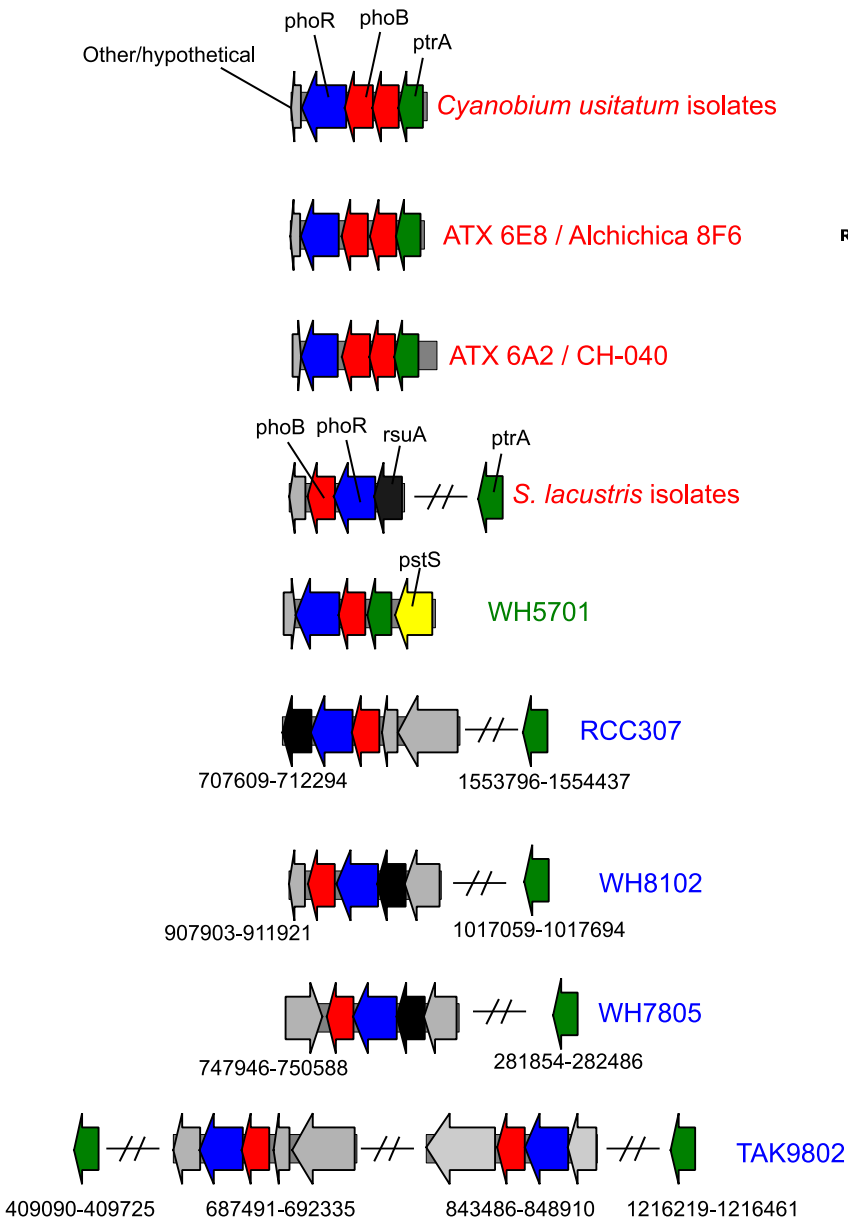

## Phylogenetic placement of freshwater isolates with 2xPhoB

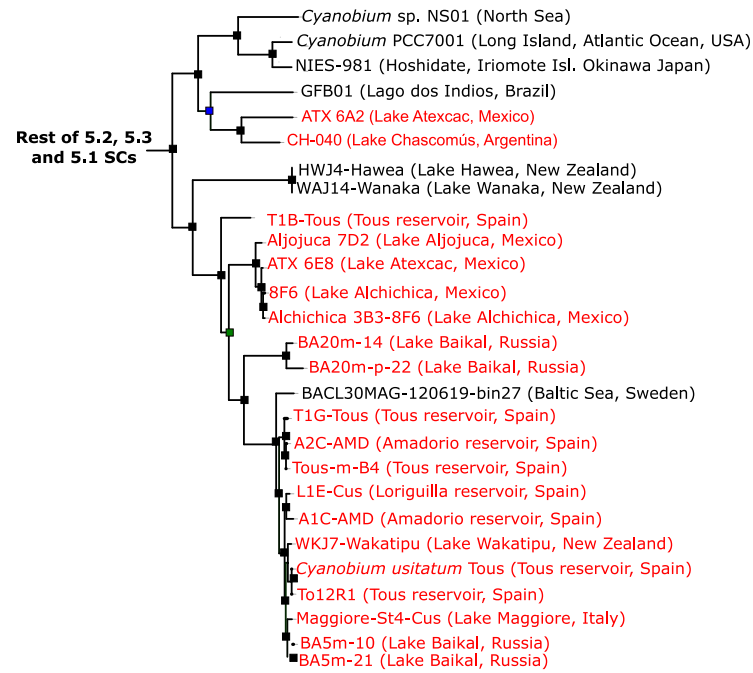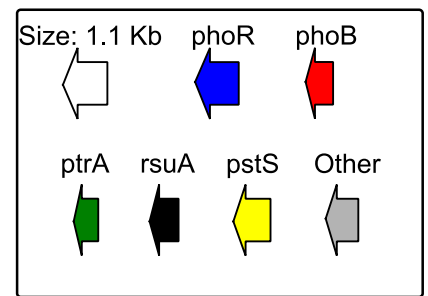

Supplement: Supplementary file 12 — Additional file 12: Fig. S3. Genomic context of the phoBR two-component system in different marine, brackish and freshwater cluster 5 picocyanobacteria. Each subunit is color coded accordingly. The right panel shows a phylogenomic tree with all those freshwater strains (colored red) possessing two copies of the phoB gene. [file 12915_2022_1379_MOESM12_ESM.pdf]

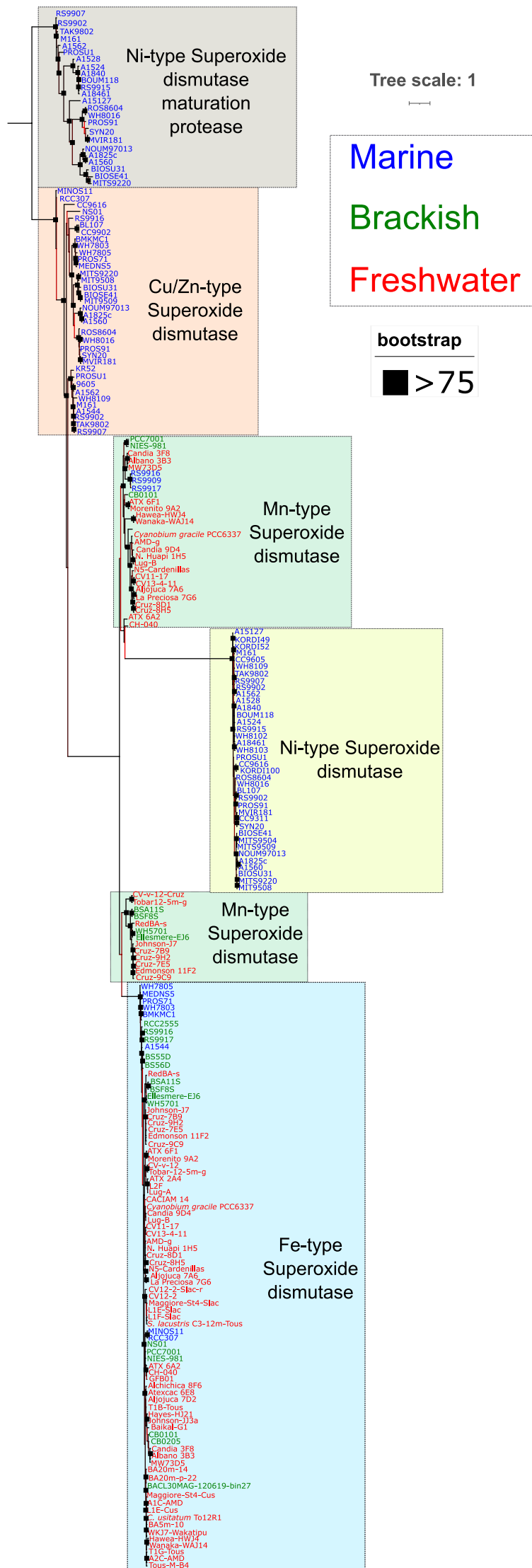

Supplement: Supplementary file 18 — Additional file 18: Fig. S4. Phylogenetic analysis of picocyanobacterial Ni/Cu/Zn/Fe/Mn superoxide dismutases. The Ni-type maturation protease from marine strains was used to root the tree. Bootstrap values >75 are shown and the habitat of each picocyanobacterial enzyme is color coded. [file 12915_2022_1379_MOESM18_ESM.pdf]

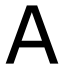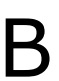

Supplement: Supplementary file 20 — Additional file 20: Fig. S5. Total number (Y axis) of A) transposases and B) integrases found in freshwater, brackish and marine picocyanobacteria. [file 12915_2022_1379_MOESM20_ESM.pdf]
